# Supplementary material for: Reversible immortalisation enables genetic correction of human muscle progenitors and engineering of next‐generation human artificial chromosomes for Duchenne muscular dystrophy
Source: EMBO Mol Med. 2017 Dec 14;10(2):254–75. doi: 10.15252/emmm.201607284 (PMC5801502; doi:10.15252/emmm.201607284)
Supplement: Supplementary file 1 — Appendix [file EMMM-10-254-s001.pdf]

Appendix

Reversible Immortalisation Enables Genetic Correction of Human Muscle Progenitors and Engineering of Next-Generation Human Artificial Chromosomes for Duchenne Muscular Dystrophy

Sara Benedetti, Narumi Uno, Hidetoshi Hoshiya, Martina Ragazzi, Giulia Ferrari, Yasuhiro Kazuki, Louise Anne Moyle, Rossana Tonlorenzi, Angelo Lombardo, Soraya Chaouch, Vincent Mouly, Marc Moore, Linda Popplewell, Kanako Kazuki, Motonobu Katoh, Luigi Naldini, George Dickson, Graziella Messina, Mitsuo Oshimura, Giulio Cossu* and Francesco Saverio Tedesco*

*Corresponding authors: giulio.cossu@manchester.ac.uk or f.s.tedesco@ucl.ac.uk

Table of Contents

- Appendix Supplementary Methods.
- Appendix Figure S1. Cre recombinase-mediated reversion of immortalisation.
- Appendix Figure S2. Engraftment of riDMD(DYSH-HAC2) mesoangioblasts in NSG mice.
- Appendix Figure S3. Myogenic differentiation and dystrophin expression in vivo upon heterotopic transplantation of DYS-HAC-containing riDMD myoblasts**.**
- Appendix Figure S4. Detailed stepwise generation of novel, synthetic multi-functional DYS-HAC4.
- Appendix Figure S5. Expression levels of native and codon-optimised human dystrophin cDNAs.
- Appendix Table S1. PCR and FISH analysis of neomycin-resistant CHO(DYS-HAC2) clones.
- Appendix Table S2. PCR and FISH analysis of neomycin-resistant A9(DYS-HAC2) clones.
- Appendix Table S3. Drug concentrations.
- Appendix Table S4. Primary antibodies.
- Appendix Table S5. PCR products.
- Appendix Table S6. List of Abbreviations for DYS-HAC3 and DYS-HAC4.
- Appendix Table S7. Primers sequences list.
- Appendix References.

Appendix Supplementary Methods

Microcell Mediated Chromosome Transfer

Microcell Mediated Chromosome Transfer (MMCT) of a novel DYS-HAC2 generated in DT40(DYS-HAC2) cells to recipient CHO cells was performed as previously described (Kazuki et al, 2010; Koi et al, 1989). For transfer of DYS-HAC2 into riDMD myoblasts, both conventional PEG-based MMCT and fusion-enhanced MMCT were performed. In the latter, fusion was enhanced by engineering CHO donor hybrids with receptors for specific surface antigen. Specifically, CHO(DYS-HAC2)-7 hybrids were engineered to express the receptor for CD13 antigen (highly expressed by riDMD myoblasts; data not shown) facilitating microcell fusion with recipient myoblasts. In both cases riDMD myoblasts were fused with microcells prepared from CHO(DYS-HAC2)-7 or anti-CD13(CHO-DYS-HAC2)-7 microcell donor hybrid, then trypsinised, sparsely seeded and cultured for fourteen days in medium with 600µg/ml of G418. DYS-HAC2-cointaining myoblasts emerged as neomycin-resistant clones. riDMD(DYS-HAC)#α, riDMD(DYS-HAC)#γ riDMD(DYS-HAC)#δ were obtained using anti-CD13(CHO-DYS-HAC2)-7, whereas riDMD(DYS-HAC)M#β was obtained with PEG-based MMCT using CHO(DYS-HAC2)-7 donor microcells.

For MMCT of DYS-HAC2 into riDMD mesoangioblasts, both A9(DYS-HAC2) and CHO(DYS-HAC2) hybrids were used as microcell donors. In both cases riDMD mesoangioblasts were fused with microcells prepared from donor hybrids A9(DYS-HAC2)-9 or CHO(DYS-HAC2)-7, respectively. One day after MMCT, they were trypsinised, sparsely plated and cultured for about fourteen days in medium with 500µg/ml of G418. DYS-HAC2-cointaining mesoangioblasts emerged as neomycin-resistant clones. riDMD(DYS-HAC)#A and riDMD(DYS-HAC)#B clones were obtained using CHO(DYS-HAC2)-7, whereas riDMD(DYS-HAC)#C and riDMD(DYS-

HAC)#D clones were obtained using A9(DYS-HAC2)-9. In the case of A9(DYS-HAC2)-mediated MMCT, polyethylene glycol (PEG) was used as fusogen between donor microcells and recipient cells (Kazuki et al, 2010), while in the case of CHO(DYS-HAC2) MMCT, CHO(DYS-HAC2)-7 donor cells were engineered to express measles virus (MV) envelope protein avoiding the use of PEG as described previously (Hiratsuka et al, 2015; Katoh et al, 2010). For the latter type of MMCT, the donor CHO(DYS-HAC2)-7 cells in 10 cm dish were co-transfected with 3μg each of linearized plasmids, pCAG-T7-F, pTNH6-H-CD13 and pTNH6-Haa-EGFR, and 2.5μg of pCMV/Bsd using Lipofectamine 2000 (Invitrogen). At 24 h after transfection, the cells were re-plated at low density and selected for about 14 days with 8μg/ml of Blasticidin (Sigma). Drug-resistant cells were recovered as a mixed population and were expanded for further MMCT using the MV as fusogen instead of PEG, reducing toxicity and increasing fusogenic potential.

Western Blot for codon optimised human dystrophin.

Plasmids expressing native (Native-hDYS) and sequence-optimised dystrophin (huDYSco) cDNAs under the transcriptional control of the cytomegalovirus (CMV) or muscle-specific Spc512 promoters were transfected into HEK293 cells. After 72h cultures were washed with ice cold PBS prior to the addition of NaCl 0.15M, HEPES 0.05M, NP-40.1%, Sodium Deoxycholate 0.5%, SDS 0.10%, EDTA 0.01M Buffer plus protease Inhibitor (Roche). Cells were then scraped, transferred to pre-chilled Eppendorf tubes and incubated on ice for 5 minutes. Following ice incubation, pellets were vortexed every 30 seconds for a further 15 minutes and then centrifuged at 13,000 rpm for 15 minutes. Protein concentrations were determined by BioRad DC Assay and denatured at 70°C for 10 minutes. 50 μg of total proteins were loaded and

separated on a 3-8% Tris- Acetate gradient gel in 1X Tris Acetate buffer. Proteins were then transferred for 2 hours at 30V on a nitrocellulose membrane. Following transfer the membrane was washed in 0.1% PBS-T, blocked in 5% in PBS-T and incubated with primary antibody overnight at 4°C. After the overnight incubation the membrane was washed 4 times with PBS-T and fluorochrome-conjugated secondary antibodies (488 Goat α Mouse and 594 Donkey α Rabbit, 1: 10,000) were incubated at room temperature for 1 hour. Fluorescent signal was detected and acquired using Odyssey imaging system.

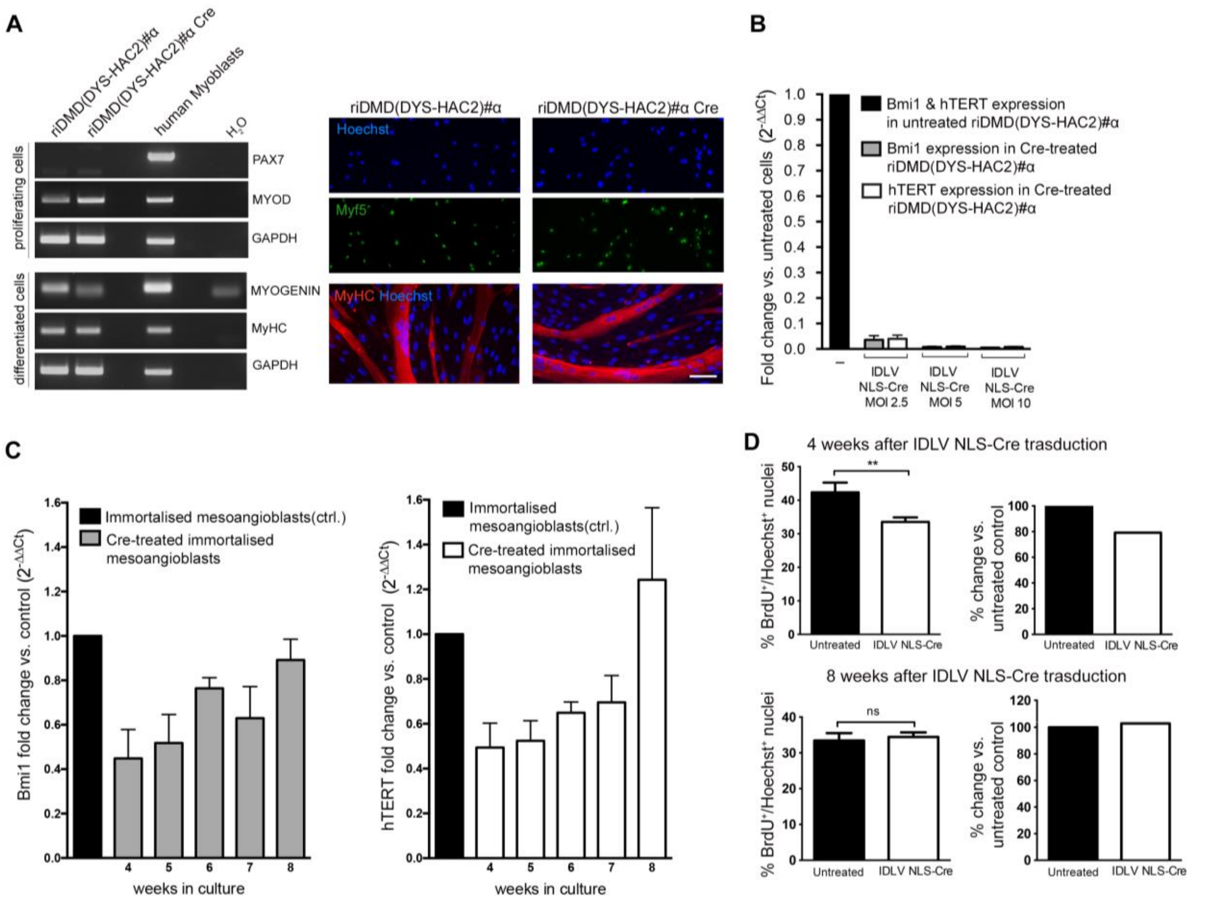

Appendix Figure S1. Cre recombinase-mediated reversion of immortalisation. (A) RT-PCR panel showing expression of PAX7, MYOD, MYOGENIN and MyHC in riDMD(DYS-HAC2)#α myoblasts before and after excision of transgenes via Integrase-Defective Lentiviral Vectors expressing a Nuclear Localization Signal Cre-recombinase (IDLV NLS-Cre). Pictures on the right side show immunofluorescence staining for MYF5 and myosin heavy chain (MyHC) before (riDMD(DYS-HAC2)#α) and after (riDMD(DYS-HAC2)#α Cre) IDLV NLS-Cre. (B) Quantitative real-time PCR of hTERT and Bmi1 expression in immortalised myoblasts (riDMD(DYS-HAC2)#α) two weeks following transduction with different multiplicity of infection (MOI; 2.5, 5 and 10) of IDLV NLS-Cre. GAPDH was used as ~~house-keeping~~ gene. Normaliser: untreated immortalised myoblasts (= 1, black bar). Data plotted as means ± SEM (N = 2-3). (C) Quantitative real-time PCR of hTERT and Bmi-1 transgenes in IDLV NLS-

Cre MOI 2.5 mesoangioblasts at different time points in culture following IDLV NLS-Cre transduction (4, 5, 6, 7 and 8 weeks). Data plotted as means ± SEM (n = 2). Normaliser: untreated immortalised myoblasts (= 1, black bar). (D) Bar graphs quantifying BrdU incorporation rate of IDLV Cre MOI 2.5 mesoangioblasts after 4 (upper graphs) and 8 weeks (bottom graphs) in culture following IDLV NLS-Cre transduction. BrdU incorporation rate was calculated as the percentage of BrdU-positive cells on the total number of nuclei after a pulse of one hour. Scale bar: 30 μm. Data plotted as means ± SEM (n = 3). **P = 0.0086, ns = 0.5178, unpaired two tailed t-test.

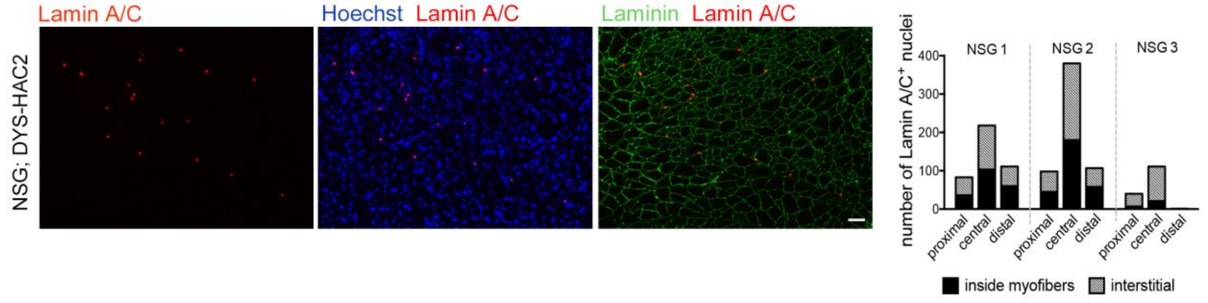

Appendix Figure S2. Engraftment of riDMD(DYSH-HAC2) mesoangioblasts in NSG mice. Low magnification pictures and additional details on quantification of the in vivo experiment reported in ~~figure~~ 6B. Immunofluorescence pictures of a frozen section of an NSG tibialis anterior cryoinjured muscle which received intramuscular injection of 10^6 MyoD-ER-transduced riDMD(DYS-HAC2) mesoangioblasts (riDMD(DYS-HAC2)#C). Red: lamin A/C; green: laminin; blue: Hoechst. Scale bar: 75 μm. Right bar graph shows mouse-specific (N = 3) quantification of histological localisation of lamin A/C positive cells from the same experiment.

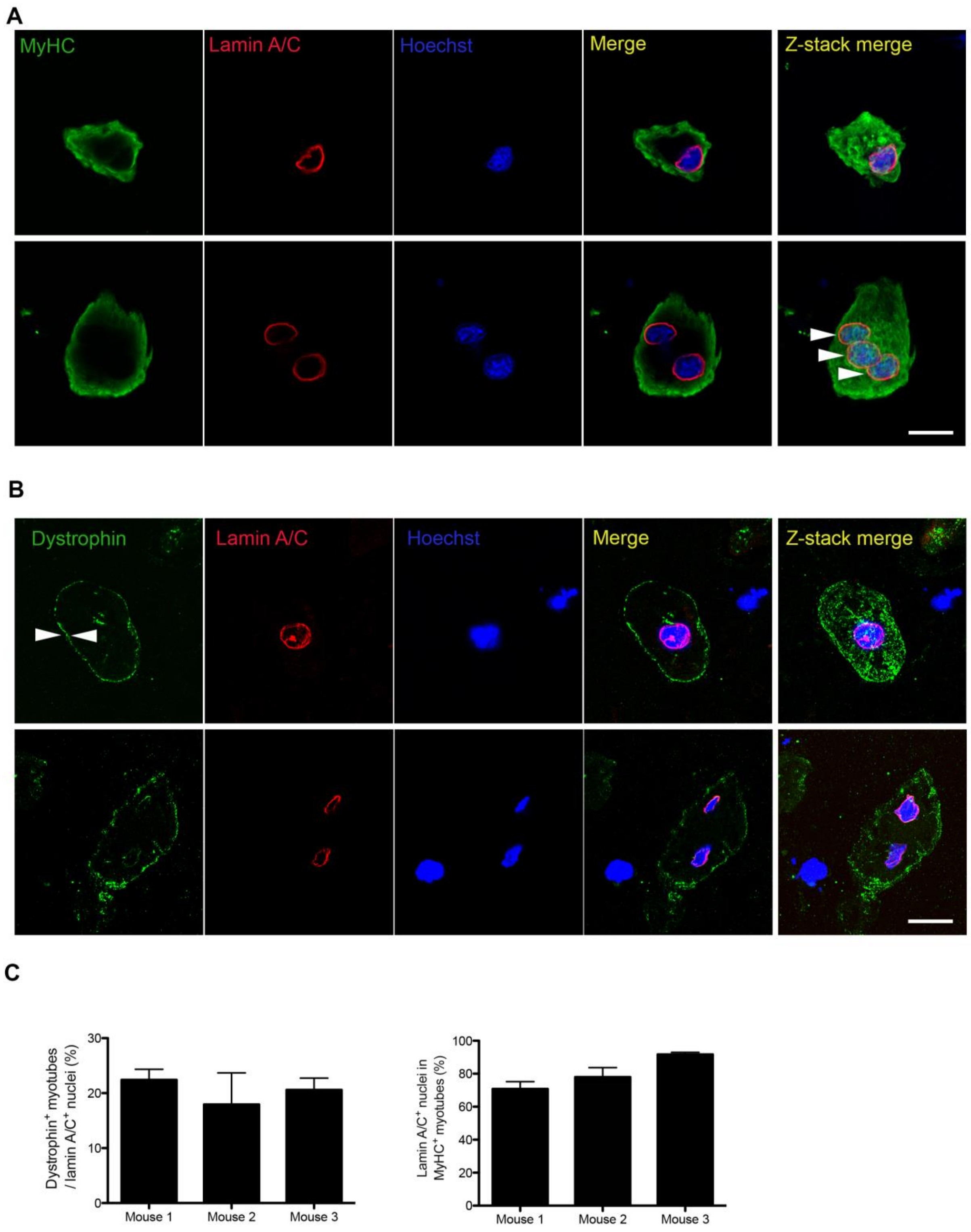

Appendix Figure S3. Myogenic differentiation and dystrophin expression in vivo upon heterotopic transplantation of DYS-HAC-containing riDMD myoblasts (full version of panel E in Fig 6). (A) Confocal microscopy pictures showing two representative myosin heavy chain-positive myotube-like structures

(green) containing human lamin A/C-positive nuclei (red) upon immunofluorescence staining of subcutaneous Matrigel plugs. 10^6 cells were suspended in cold undiluted Matrigel and were injected subcutaneously two weeks earlier in immunodeficient NSG mice. Arrowheads highlight nuclei within a multi-nucleated, non-conventional myotube. (B) Confocal microscopy pictures as in (A) showing two representative dystrophin-positive myotube-like structures. Arrowheads highlight dystrophin peri-membrane staining pattern. (C) Quantification of the staining shown in (A) and (B). Four mice were heterotopically transplanted, all showed engraftment and Matrigel plugs from three randomly selected animals were quantified. A minimum of 130 human nuclei per plug were counted (in 9 randomly selected high-power fields). Scale bars: 15μm.

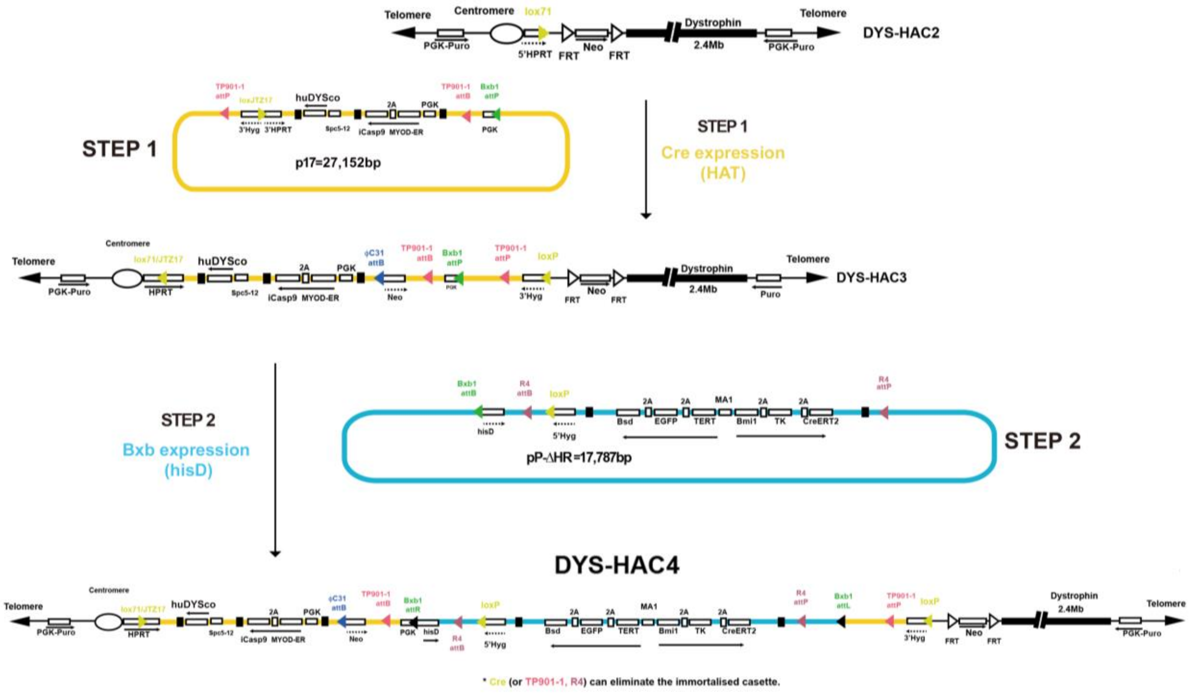

Appendix Figure S4. Detailed stepwise generation of novel, synthetic multi-functional DYS-HAC4. DYS-HAC4 was engineered by sequential insertion of plasmids p17 (yellow line) and pP-ΔHR (blue line) into DYS-HAC2 (black line; Fig 1A), generating an intermediate DYS-HAC named DYS-HAC3. A complete list of abbreviations is described in Appendix Table S6. Black arrows indicate direction of expression and black boxes indicate insulators.

<u>STEP1: construction of DYS-HAC3 by insertion of p17 into DYS-HAC2.</u> p17 plasmid was synthesised to carry two cassettes: 1) a codon optimised human dystrophin cDNA (huDYSco) under the control of the Spc5-12 muscle-specific promoter to increase dystrophin gene dosage; 2) an inducible Caspase9 (iCasp9) and MYOD-ER for controllable cell death (safety system) and myogenic differentiation. MYOD-ER and iCasp9 were linked with 2A sequence and controlled by a PGK promoter. Insulators were set on the both sides of each cassette. 5' HPRT lox71 site previously cloned on DYS-HAC2 (Fig 1A) and a loxJTZ173'HPRT cloned

on p17 plasmid, were used for insertion of p17 (yellow line) by Cre-lox71/loxJZT17 recombination, resulting in HPRT gene reconstitution (Araki et al, 2010).

STEP2: DYS-HAC4 construction by insertion of pP-ΔHR into DYS-HAC3 in order to add hTERT and Bmi1 immortalising cassette. The pP-ΔHR was inserted into DYS-HAC3 by site-specific recombination between Bxb1 attP on DYS-HAC3 and Bxb1 attB on pP-ΔHR following Bxb1 expression. The immortalising cassette included TERT, EGFP and Bsd resistance genes, all linked with 2As; in the same way Bmi1, TK and CreERT2 were also linked together with 2A elements. These two cDNAs were cloned under the control of the MA1bi-directional promoter, minimising cassette length. The immortalising cassette was cloned between two loxP sites for self-elimination via CreERT2 following tamoxifen treatment or via other integration systems including R4 attB/attP (magenta triangles) and TP901-1 attB/attP (pink triangles) (restoring hygromycin transcription and resistance).

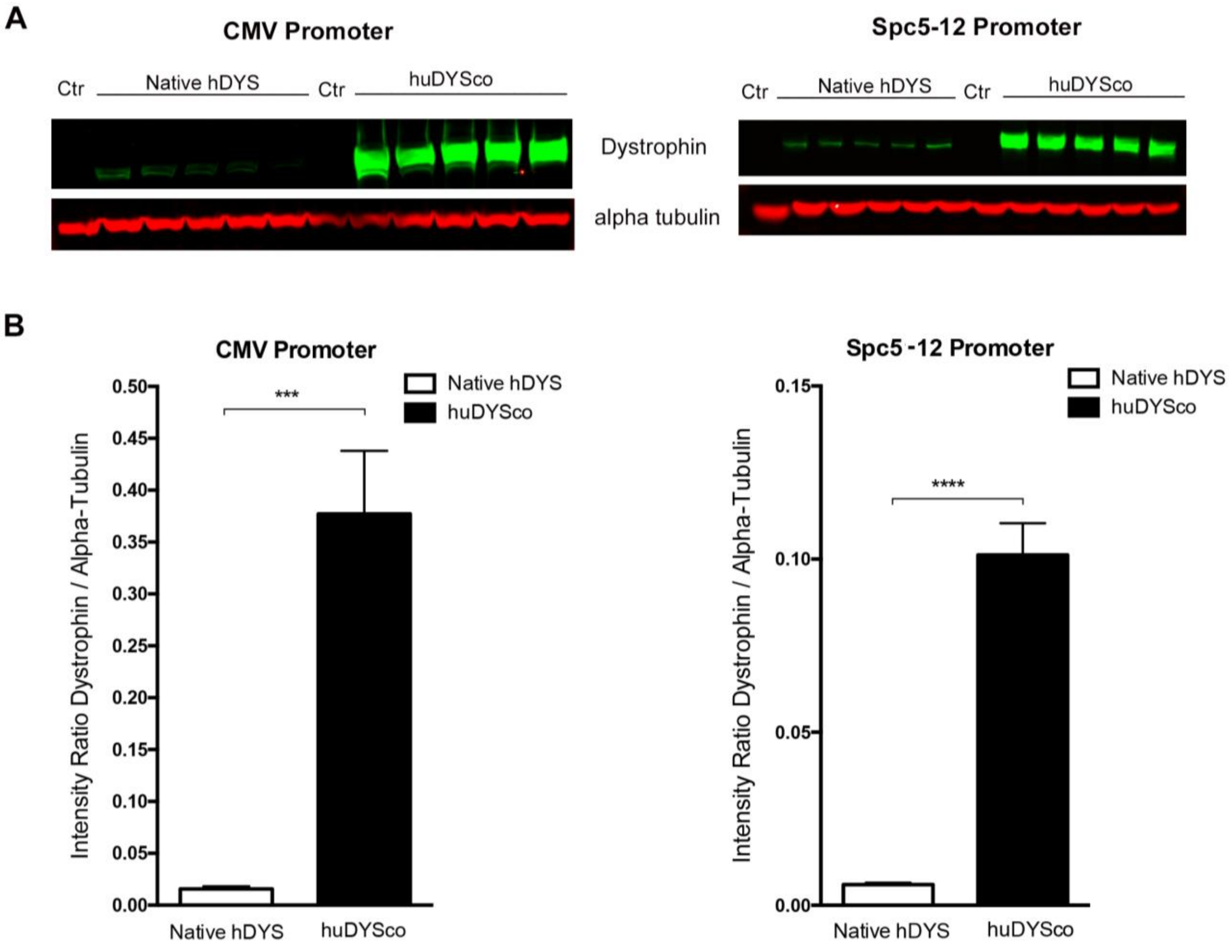

Appendix Figure S5. Expression levels of native and codon-optimised human dystrophin cDNAs. A) Western Blot showing dystrophin expression levels (green signal) in Hek293T cells previously transfected with plasmids carrying native (Native hDYS, n = 5) and sequence-optimised (huDYSco, n = 5) dystrophin cDNAs, under the transcriptional control of the cytomegalovirus (CMV; left) or synthetic Spc5-12 promoters (right). Alpha-tubulin was used as normaliser (red signal). Ctr: untransfected Hek293T cell lysate used as negative control for dystrophin expression. (B) Graph bars showing quantification of dystrophin expression of (A). Dystrophin was quantified in relation to alpha-tubulin expression. Data are plotted as mean ± SEM (n = 5). *** $P = 0.0003$, **** $P < 0.0001$, unpaired two tailed t-test.

Appendix Table S1. PCR and FISH analysis of neomycin-resistant CHO(DYS-HAC2) clones.

	Primers for HAC regions		*Primers for human dystrophin*							*FISH*
Clones	Puro	sk23/DMDt5R	DYS 1L/1R	DYS 4L/4R	DYS 5L/5R	DYS 6L/6R	DYS 7L/7R	DYS 7L/8R	DYS 8L/8R	Episomal HAC
CHO(DYS-HAC2)-1	+	NT	-	NT	NT	NT	NT	NT	NT	NT
CHO(DYS-HAC2)-2	+	NT	-	NT	NT	NT	NT	NT	NT	NT
CHO(DYS-HAC2)-3	+	NT	+	+	-	+	+	-	+	NT
CHO(DYS-HAC2)-4	+	NT	+	+	-	+	+	-	+	NT
CHO(DYS-HAC2)-5	-	NT	NT	NT	NT	NT	NT	NT	NT	NT
CHO(DYS-HAC2)-6	+	+	+	+	+	+	+	+	+	-
CHO(DYS-HAC2)-7	+	+	+	+	+	+	+	+	+	+
CHO(DYS-HAC2)-8	+	NT	-	NT	NT	NT	NT	NT	NT	NT
CHO(DYS-HAC2)-9	-	NT	NT	NT	NT	NT	NT	NT	NT	NT
CHO(DYS-HAC2)-10	+	+	+	+	+	+	+	+	+	+
CHO(DYS-HAC2)-11	+	+	+	+	+	+	+	+	+	+
CHO(DYS-HAC2)-12	+	+	+	+	+	+	+	+	+	+
CHO(DYS-HAC2)-13	+	NT	+	+	+	+	+	-	+	NT
CHO(DYS-HAC2)-14	-	NT	NT	NT	NT	NT	NT	NT	NT	NT
CHO(DYS-HAC2)-15	+	NT	-	NT	NT	NT	NT	NT	NT	NT
CHO(DYS-HAC2)-16	+	NT	-	NT	NT	NT	NT	NT	NT	NT
CHO(DYS-HAC2)-17	+	NT	-	NT	NT	NT	NT	NT	NT	NT
CHO(DYS-HAC2)-18	+	NT	-	NT	NT	NT	NT	NT	NT	NT
CHO(DYS-HAC2)-19	+	NT	-	NT	NT	NT	NT	NT	NT	NT
CHO(DYS-HAC2)-20	-	NT	NT	NT	NT	NT	NT	NT	NT	NT
CHO hprt-/- (parental)	-	-	-	-	-	-	-	-	-	−
DT40(DYS-HAC2)-1	+	+	+	+	+	+	+	+	+	+
Positive clones	5/20									4/5

Twenty CHO clones have been initially selected among 111 G418 (neomycin)-resistant ones, which emerged after serial-dilution cloning. All selected clones were analysed by PCR for HAC structure and human dystrophin exons and 5 out of 20 were positive. PCR-positive clones were then tested by FISH to verify presence of DYS-HAC2. Four out of 5 showed an episomal single copy of DYS-HAC2. DYS-HAC DT40(DYS-HAC2)-1: positive control; CHO hprt-/- (parental): negative control; +, positive; −, negative; NT, not tested.

Appendix Table S2. PCR and FISH analysis of neomycin-resistant A9(DYS-HAC2) clones.

	Primers for different HAC regions		*Primers for different human dystrophin regions*						*FISH*
Clones	Puro	SK23/DMDt5R	DYS 3L/3R	DYS 4L/4R	DYS 5L/5R	DYS 6L/6R	DYS 7L/7R	DYS 8L/8R	Episomal HAC
A9(DYS-HAC2)-1	-	+	+	+	+	+	+	+	NT
A9(DYS-HAC2)-2	+	+	+	+	+	+	+	+	+
A9(DYS-HAC2)-3	+	-	+	+	+	-	-	-	NT
A9(DYS-HAC2)-4	+	-	+	+	+	-	-	-	NT
A9(DYS-HAC2)-5	+	-	+	+	+	-	-	-	NT
A9(DYS-HAC2)-6	+	-	+	+	+	-	-	-	NT
A9(DYS-HAC2)-7	+	-	+	+	+	-	-	-	NT
A9(DYS-HAC2)-8	+	-	+	+	-	-	-	-	NT
A9(DYS-HAC2)-9	+	+	+	+	+	+	+	+	+
A9(DYS-HAC2)-10	+	+	+	-	-	+	+	+	NT
A9(DYS-HAC2)-11	+	+	+	+	+	+	+	+	NT
A9(DYS-HAC2)-12	+	-	+	+	+	-	-	-	NT
A9(DYS-HAC2)-13	+	-	+	+	+	-	-	-	NT
A9(DYS-HAC2)-14	+	-	+	+	+	-	-	-	NT
A9(DYS-HAC2)-15	+	+	+	+	+	+	-	-	NT
A9(DYS-HAC2)-16	+	-	+	+	+	-	-	-	NT
A9(DYS-HAC2)-17	+	-	+	+	+	-	-	-	NT
A9(DYS-HAC2)-18	+	-	+	+	+	-	-	-	NT
A9(DYS-HAC2)-19	+	-	+	+	+	-	-	-	NT
A9(DYS-HAC2)-20	+	-	+	+	+	-	-	-	NT
A9(DYS-HAC2)-21	+	-	+	+	+	-	-	-	NT
A9(DYS-HAC2)-22	+	+	+	+	+	-	-	-	NT
A9(DYS-HAC2)-23	+	+	+	+	+	+	+	+	-
A9(DYS-HAC2)-24	+	-	+	+	+	-	-	-	NT
A9(DYS-HAC2)-25	+	-	+	+	+	-	-	-	NT
A9(DYS-HAC2)-26	+	+	+	+	+	+	+	+	+
A9(DYS-HAC2)-27	+	+	+	+	+	+	+	+	-
A9 (parental)	-	-	-	-	-	-	-	-	-
CHO(DYS-HAC2)-7	+	+	+	+	+	+	+	+	+
Positive clones				5/27					3/5

Twenty-seven A9 clones have been randomly selected among neomycin-resistant clones and were analysed by PCR for different HAC and dystrophin exon regions. 5 out of 27 PCR-positive clones were tested by FISH to detect DYS-HAC2. 3 out 5 had a single and episomal copy of DYS-HAC2 (highlighted in grey). CHO (DYS-HAC2)-7: positive control; A9 (parental): negative control; +: positive; −: negative; NT: not tested

Appendix Table S3. Drug concentrations.

Cell type	Colcemid		G418
	MMTC	*FISH/Karyotype*	*Selection*
DT40(DYSH-HAC2)	0.25 µg/ml, 12 hs	0.05 µg/ml, 40 min	1.5 mg/ml
A9(DYSH-HAC2)-10	0.05 µg/ml, 24 hs	0.05 µg/ml, 2 hs	0.8 mg/ml
CHO(DYSH-HAC2)7	0.1 µg/ml, 72 hs	0.2 µg/ml, 2 hs	0.8 mg/ml
Human mesoangioblasts	NA	0.1-0.2 µg/ml, 3 hs - O/N	0.5 mg/ml
Human myoblasts	NA	0.025 µg/ml, 3 hs	0.6 mg/ml

NA: not applicable; hs: hours.

Appendix Table S4. Primary Antibodies.

Antibody	Company	Dilution
Mouse anti-dystrophin Dys1	Novocastra	1:50 (IF)
Mouse anti-dystrophin Dys2	Novocastra	1:50 (IF)
Mouse anti-dystrophin Dys3	Novocastra	1:50 (IF)
Rabbit anti-dystrophin (RB-9024P1)	Fisher	1:500/1:1000 (IF)
Mouse anti-MyHC MF20	Developmental Studies Hybridoma Bank	1:5 (IF), 1:10 (WB)
Mouse anti-laminA/C	Novocastra	1:500 (IF)
Rabbit anti-laminin	Sigma-Aldrich	1:300 (IF)
Rabbit anti-GFP	Chicken	1:300 (IF)
Chicken anti-GFP	Millipore	1:300 (IF)
Mouse Gapdh	Sigma-Aldrich	1.5000 (WB)
Mouse Bmi-1	Upstate	1:100 (WB)
Rabbit Myf5	Santa Cruz	1:100 (IF)
Mouse MANDYS106	MDA Monoclonal Antibody Resource	1: 20 (WB)
Mouse MANEX1011C	MDA Monoclonal Antibody Resource	1: 50 (WB)
Rabbit Tubulin	Abcam	1: 2500 (WB)

WB: western blot; IF: immunofluorescence.

Appendix Table S5. PCR products.

Primers	Amplicon name*	Product size (bp)
21CenG1/NeoR	5'	6526 bp
NeoF/DloxP3L	3'	3845 bp
NeoF/NeoR	Neo	391 bp
Bsd2F/BsdR	Bsd	399 bp
EGFPL/EGFPR	EGFP	479 bp
DYS1L/DYS1R	DYS1L/DYS1R	168 bp
DYS1L/DYS2R	DYS1L/DYS2R	331 bp
DYS3L/DYS3R	DYS3L/DYS3R	163 bp
DYS4L/DYS4R	Exon 23	129 bp
DYS5L/DYS5R	DYS5L/DYS5R	132 bp
DYS6L/DYS6R	DYS6L/DYS6R	170 bp
DYS6L/DYS7R	DYS6L/DYS7R	318 bp
DYS7L/DYS7R	DYS7L/DYS7R	151 bp
DYS7L/DYS8R	DYS7L/DYS8R	706 bp
DYS8L/DYS8R	DYS8L/DYS8R	155 bp
sk23/DMDt5R	SK23DMD5t	Approximately 8.8kbp
PuroI/pce1L	Puro	8289bp
Furin-L/Furin-R	Furin	Approximately 280bp
DMDEX04F/DMDEX04R	Exon 4	233 bp
DMDEX05F/DMDEX05R	Exon 5	261 bp
DMDEX06F/DMDEX06R	Exon 6	335 bp
DMDEX07F/DMDEX07R	Exon 7	269 bp
DMDEX08F/DMDEX84R	Exon 8	343 bp
DMDEX1-21F/DMDEX4-428R	Exon 1 - Exon 4	408 bp
DMDEX3-305F/DMDEX6-726R	Exon 3 - Exon 6	422 bp
DMDEX6-621F/DMDEX9-1137R	Exon 6 - Exon 9	517 bp
CMVhTERT FW/hTERT REV	hTERT (transgene)	110 bp
CMVBmi1 FW/Bmi1 REV	Bmi1 (transgene)	144 bp
hTERT FW/hTERT REV	hTERT (cDNA)	196 bp
Bmi1 FW/Bmi1 REV	Bmi1 (cDNA)	104 bp
DYS11L/DYS10R	Exon 46	67 bp
DYS2L/DYS2R	Exon 50	177 bp
TRANS L1/TRANS R1	HPRT	398 bp
HPRT6L/hDyscoR6	HPRT6L/huDYScoR6	2084 bp

hDyscoF1/hDyscoR1	hDyscoF1/R1	2237 bp
iCasp9F1/iCasp9R2	iCasp9F1/R2	1035 bp
Bxb1attBF1/HygF2	Bxb1attBF1/HygF2	3297 bp
HygF1/HR2.6R1	HygF1/HR2.6R1	2176 bp
TP901-1attBF1/hisDR1	TP901-1attBF1/hisDR1	977 bp
hTERT2F/hTERT2R	hTERT2F/2R	1201 bp
hTERT3R/Bmi2R	hTERT3R/Bmi2R	2260 bp
hDyscoF5/hDyscoR5	hDyscoF5/R5	2505 bp
hDyscoF6/spc512R1	hDyscoF6/spc512R1	2071 bp
hDyscoF2/hDyscoR2	hDyscoF2/R2	2274 bp
hMYODERT2F1/PhiC31attBR1	hMYODERT2F1/PhiC31attBR1	2023 bp
PhiC31attBF1-2/hisDR2	PhiC31 F1-2/hisDR2	2690 bp
hisDF1/hisDR2	hisDF1/DR2	667 bp
DYS cDNA rt-F1/DYS cDNA rt-R1	DYS cDNA rt-F1/R1	211 bp
DYS cDNA rt-F2/DYS cDNA rt-R2	DYS cDNA rt-F2/R2	230 bp
DYS cDNA rt-F3/DYS cDNA rt-R3	DYS cDNA rt-F3/R3	230 bp
hTERT rt-F1/hTERT rt-R1	hTERT rt-F1/R1	176 bp
MYODER rt-F1/MYODER rt-R1	MYODERT2-iCasp9	169 bp
Bmi1 rt-F1/TK rt-R1	Bmi1-TK	234 bp
PAX7 FW/PAX7 REV	PAX7	390 bp
MYOD FW/MYOD REV	MYOD	255 bp
MYOGENIN FW7MYOGENIN REV	MYOGENIN	105 bp
MyHC FW/MyHC REV	MyHC	102 bp
DYS49-50 F/DYS50 R	DYS	113 bp
[†]Gapdh F/Gapdh R	Gapdh	237 bp
[‡]Gapdh F/Gapdh R	Gapdh	123 bp
[#]Gapdh F/Gapdh R	Gapdh	Approximately 700 bp
h actinin F/h actinin R	hActinin	996 bp
NV1 F/ NV1 R	NV1	Approximately 150 bp

*Name used in the main text, legends and figures. FW/F: forward; REV/R: reverse. Neo: neomycin; Bsd: blasticidin; Puro: puromycin. PCR conditions (i.e. temperatures, master mix composition, primers concentrations) were key for amplification, especially larger amplicons, and are available upon request. [†]Set of primers used for Figures 3B,C, 5B and S1A; [‡]set of primers used for Figures 4E and 6A; [#]set of primers used for Figure 7F;

Appendix Table S6. List of Abbreviations for DYS-HAC3 and DYS-HAC4.

Abbreviation	Full name	Length	Function in DYS-HAC4 construction
PGK	Phosphoglycerate kinase promoter	508bp	Promoter for constitutive gene expression
Neo	Neomycin	795bp	Selection marker inducing resistance to G418
Puro	Puromycin	600bp	Selection marker inducing resistance to puromycin
5' HPRT	A fragment including exon 1 and 2 of phosphoribosyltransferase (HPRT)	133bp	Reconstituted HPRT gene confers HAT resistance to HPRT KO cell line.
3' HPRT	A fragment including exon 3-9 of phosphoribosyltransferase (HPRT)	522bp	
Lox71	Locus of X (cross) 71	34bp	Mutated loxP sequences for Cre-lox71/JTZ17 recombination system for p17
LoxJTZ17	Locus of X (cross) JTZ17	34bp	
FRT	Flippase recognition target	34bp	Elimination of Neo resistance gene
HAT	Hypoxanthine-Aminopterin-Thymidine	N/A	Selection drug for HPRT expressing cells, indicating integration of p17 into DYS-HAC2 and generation of DYS-HAC3 -Step 1 Appendix Figure S2-
Dystrophin	Dystrophin locus	Approx 2.4Mb	Complete genetic correction for DMD
TP901-1 attP	Phage TP901-1 of *Lactococcus lactis* subsp. *Cremoris,* phage attachment site	69bp	Back-up system for elimination of the immortalising cassette via TP901-1 integrase expression
TP901-1 attB	Phage TP901-1 of *Lactococcus lactis* subsp. *Cremoris,* bacterial attachment site	69bp	Back-up system for elimination of the immortalising cassette via TP901-1 integrase expression
Ins	Insulator HS4	1196bp	Stable expression of neighbouring genes/cDNAs
huDYSco	Codon-optimised human Dystrophin cDNA	11061bp	Codon-optimised human dystrophin cDNA to increase dystrophin expression level
Spc5-12	Synthetic promoter c5-12	346bp	Synthetic muscle specific promoter
iCasp9	Inducible Caspase-9	1239bp	A safeguard system to induce (DYS-HAC4) containing cell apoptosis following dimerizing drug AP1903
2A	2A Peptide Derived from Porcine Teschovirus-1	66bp	Element linking up-stream and down-stream genes generating fusion proteins
MYOD-ER	Human myogenic differentiation 1 protein, fused with human estrogen receptor T2	1908bp	Induction of myogenic differentiation following tamoxifen treatment

Bxb1 attP	Bxb1 bacteriophage, phage attachment site	77bp	Insertion sites for pP-ΔHR into DYS-HAC3 by Bxb1 integrase expression
Bxb1 attB	Bxb1 bacteriophage, bacterial attachment site	77bp	
hisD	Histidinol dehydrogenase	1305bp	Selection marker for L-histidinol dihydrochrorid expressing cells, indicating integration of pP-ΔHR into DYS-HAC3 and generation of DYS-HAC4 -Step 2 Appendix Figure S2-
3'Hyg	3' fragment of Hygromycin gene	1026bp	Reconstituted Hygromycin gene
5'Hyg	5' fragment of Hygromycin gene (PGK promoter)	508bp	
LoxP	Locus of X(cross) in Phage	34bp	Elimination of the immortalising cassette via Cre expression
Bsd	Blasticidin S-resistance gene	396bp	Selection marker for insertion of the immortalising cassette
EGFP	Enhanced green fluorescence protein	714bp	EGFP expression will indicate proper insertion of immortalising cassette
hTERT	Human Telomere Reverse Transcriptase	3396bp	Immortalisation of expressing cell via telomere elongation
MA1	Bidirectional promoter, combined with mini CMV and PGK promoters	109+516bp	Promoter for constitutive gene expression of hTERT-EGFP-Bsd and Bmi1-TK-CreERT2
Bmi1	B cell-specific Moloney murine leukemia virus integration site 1 (Polycomb gene)	972bp	Immortalisation of expressing cells via inhibition of p16 -mediated senescence pathway
TK	HSV-Tk mutant 2	1125bp	Elimination of cells containing the immortalising cassette following ganciclovir expression
CreERT2	a Type I topoisomerase from bacteriophage P1, fused with ERT2	1980bp	Elimination of the immortalising cassette induced by tamoxifen treatment
R4 attB	R4 phage lysogenizes *S. parvulus,* bacterial attachment site	57bp	Back-up system for elimination of the immortalising cassette via R4 integrase Expression
R4 attP	R4 phage lysogenizes *S. parvulus,* phage attachment site	57bp	Back-up system for elimination of the immortalising cassette via R4 integrase Expression
phiC31 att B	Streptomyces phage (phi)C31, bacterial attachment site	273bp	Sequential insertion system for circular vector

N/A: not applicable. Approx: approximately.

Appendix Table S7. Primers sequences list.

- NeoF: 5’- CACAACAGACAATCGGCTGCTCT-3’
- NeoR: 5'- TGATCGACAAGACCGGCTTCCA-3'
- DloxP3L: 5'- GCATGGGGGAGGAGAGAAGAGAGATGTA-3'
- 21CenG1L: 5'- GTGAAGGCATTGCCAGTGTTTTCTTCTG-3'
- hCMV586: 5'- CGTAACAACTCCGCCCCATT-3'
- Bsd2F: 5’-ATGGCCAAGCCTTTGTCTC-3’
- Bsd2R: 5’-TTAGCCCTCCCACACATAAC-3’
- EGFPL: 5’-CCTGAAGTTCATCTGCACCA-3’
- EGFPR: 5’-TGCTCAGGTAGTGGTTGTCG-3’
- DYS1L: 5’- TGCTCTGGCTCATGTGTTTGC-3’
- DYS1R: 5’-AGCTCCCCTTTCGCATGATTC-3’
- DYS2L: 5’- CGAAAGGGGAGCTGTTGGAAT-3’
- DYS2R: 5’-CCATGCCAGCTGTTTTTCCTG-3’
- DYS3L: 5’- AACAACTGAACAGCCGGTGGA-3’
- DYS3R: 5’- GGGGTGGTGGGTTGGATTTT-3’
- DYS4L: 5’-GCAAGAGCAACAAAGTGGCCTA-3’
- DYS4R: 5’- AGCTTCTTCCAGCGTCCCTCA-3’
- DYS5L: 5’- ACCTTCAGAACCGGAGGCAAC-3’
- DYS5R: 5’- AGGGACCCTCCTTCCATGACTC-3’
- DYS6L: 5’- TGGAACGCATTTTGGGTTGTT-3’
- DYS6R: 5’- AAAACAATGCGCTGCCTCAAA-3’
- DYS7L: 5’-TTTGCATCCTTTTGGCGTGAT-3’
- DYS7R: 5’- AAACTCAAGCCTGCCCCACTC-3’
- DYS8L: 5’- GCTGCTAGCAATGCCACGATT-3’

- DYS8R: 5’- GGATGGGCTGGGAATCCATAG-3’
- sk23: 5’- GGCCGCTCTAGAACTAGTGGATC -3’
- DMDt5R: 5’- CTGAGCCCTCACCAGAATCACCTTGATA -3’
- PuroI: 5’- GAGCTGCAAGAACTCTTCCTCACG-3’
- pce1L: 5’- ACTGCTGCCATGCAGACAGTTGTGCTTT-3’
- Furin-L: 5’- ACTCAGAGATCCACTGCACCAGGATCCAAGGGAGG -3’
- Furin-R: 5’- CCGCTCGAGGCGGCTACACCACAGACACCATTGTT GGCTACTGCTGCC -3’
- DMDEX04F: 5’- TTGTCGGTCTCTCTGCTGGTCAGTG -3’
- DMDEX04R: 5’- CCAAAGCCCTCACTCAAAC -3’
- DMDEX05F: 5’- CAACTAGGCATTTGGTCTC -3’
- DMDEX05R: 5’- TTGTTTCACACGTCAAGGG -3’
- DMDEX06F: 5’- TGGTTCTTGCTCAAGGAATG -3’
- DMDEX06R: 5’- TGGGGAAAAATATGTCATCAG -3’
- DMDEX07F: 5’- CTATGGGCATTGGTTGTC -3’
- DMDEX07R: 5’- AAAAGCAGTGGTAGTCCAG -3’
- DMDEX08F: 5’- TCGTCTTCCTTTAACTTTG -3’
- DMDEX08R: 5’- TCTTGAATAGTAGCTGTCC -3’
- DMDEX1-21F 5’- CTACAGGACTCAGATCTGGG -3’
- DMDEX4-428R 5’- GGGCATGAACTCTTGTGGAT -3’
- DMDEX3-305F 5’- GGGAAGCAGCATATTGAGAA -3’
- DMD EX6-726R 5’- ATGAGAGCATTCAAAGCCAG -3’
- DMDEX6-621F 5’- AGATTCTCCTGAGCTGGGTC -3’
- DMDEX9-1137R 5’- GAGGTGGTGACATAAGCAGC -3’
- CMV FW hTERT (transgene) 5’-TAGGCGTGTACGGTGGAGG-3’

- hTERT REV (transgene) 5’-AGAGTCGGTGTCTTCTATGGAG-3’
- CMV FW Bmi1 (transgene) 5’-GGAGACGCCATCCACGCTGTTT-3’
- Bmi1 REV (transgene) 5’-CACACAGGACACACATTAAGTGGGG-3’
- hTERT FW (cDNA) 5’-CAGGGGCAAGTCCTACGTCC-3’
- hTERT REV (cDNA) 5’-CCTGAGGAAGGTTTTCGCGT-3’
- Bmi1 FW (cDNA) 5’-CTGGAGAAGAAATGGCCCACTA-3’
- Bmi1 REV (cDNA) 5’-GCTCTCCAGCATTCGTCAGT-3’
- DYS11L: 5’- TGTTTTATGGTTGGAGGAAGC-3’
- DYS10R: 5’- TTGCTGCTCTTTTCCAGGTT-3’
- DYS12F: 5’- GGCCGGGTTGGTAATATTCT-3’
- DYS12R: 5’- TTGCTTTGTTTTTCCATGCT-3’
- TRANS L1: 5’-GGAGGCCATAAACAAGAAGAC-3’
- TRANS R1 5’-CCCCTTGACCCAGAAATTCCA-3’
- HPRT6L: 5’-GCATCTAAGAAGTTTTGTTCTGTCC-3’
- hDyscoR6: 5’-CTCCCTGGAAAGCGAGGAAA-3’
- hDyscoF1: 5’-ACTTCTTCCAGTCCTGTGCT-3’
- hDyscoR1: 5’-GAGTGGGAGAAGCTGAACCT-3’
- hDyscoF2: 5’-TGTGGTCAGGCAGTTGATGA-3’
- hDyscoR2: 5’-GTGAAGCGCAAACTGGAAGA-3’
- hDyscoF5: 5’-TTTCTGCTGGGCCTCTTCTT-3’
- hDyscoR5: 5’-AAAGACCAGTTCCACACCCA-3’
- hDyscoF6: 5’-GTCCACCGGCAGATGTTG-3’
- spc512R1: 5’-ACCATCCTCACGACACCCA-3’
- iCasp9F1: 5’-GTCGAGTGCGTAGTCTGGTA-3’
- iCasp9R2: 5’-ATGAGTGTGGGTCAGAGAGC-3’

- hMYODERT2F1: 5'-TGCTCTTCGGGTTTCAGGAG-3'
- PhiC31attBR1: 5'-GGATCAACTACCGCCACCT-3'
- HygF1: 5'-AGCTATTTACCCGCAGGACA-3'
- HR2.6R1: 5'-TAGGTGGAGAGGTTTCAAGGG-3'
- TP901-1attBF1: 5'-TTGCCAACACAATTAACATCTCA-3'
- hisDR1: 5'-TTTCCACATCTACAGGCGGT-3'
- PhiC31attBF1-2: 5'-CGTACTCCACCTCACCCATC-3'
- hisDR2: 5'-GCAGGTCAGAAGCAACGAAA-3'
- hisDF1: 5'- ACGGTCAGCGATATTCTGGA-3'
- hTERT2R: 5'-TCTTGAAGTCTGAGGGCAGT-3'
- hTERT2F: 5'-ATCGCCAGCATCATCAAACC-3'
- hTERT3R: 5'-ACACCTGGTAGGCGCAG-3'
- Bmi2R: 5'-TCGAGGTCTACTGGCAAAGG-3'
- Bxb1attBF1: 5'-GTCGGCCGGCTTGTCG-3'
- HygF2: 5'-ATATGCGCGATTGCTGATCC-3'
- DYS cDNA rt-F1: 5'-AGAGAACCAGCAGCCCCAAG-3'
- DYS CDNA rT-R1: 5'-GACAGCAGCCAGGACAGCAC-3'
- DYS CDNA rT-F2: 5'-GCTGAAATGCCTGGACGACA-3'
- DYS CDNA rT-R2: 5'-ACTGGGCGAACTTGCTCTCG-3'
- DYS CDNA rT-F3: 5'-CGACACCCACAGCAGAATCG-3'
- DYS CDNA rT-R3: 5'-CGCCTCTTTCCTCGCTTTCC-3'
- hTERT rT-F1: 5'-CCATCCTCTCCACGCTGCTC-3'
- hTERT rT-R1: 5'-CTCAGGGACACCTCGGACCA-3'
- MYODER rT-F1: 5'-CCTCTCCCACATCAGGCACA-3'
- MYODER rT-R1: 5'-GCTTTGGTCCGTCTCCTCCA-3'

- TK rt-F1: 5’-TGGCAAAGGAAGATTGGTGGTT-3’
- TK rt-R1: 5’-GATGGGGAAAACCACCACCA-3’
- PAX7 FW: 5’-CAAGATTCTTTGCCGCTACC-3’
- PAX7 REV: 5’-TTCAGTGGGAGGTCAGGTTC-3’
- MYOD FW: 5’-CACTCAAGCGCTGCACGTCG-3’
- MYOD FW: 5’-GGCCGCTGTAGTCCATCATGC-3’
- MYOGENIN FW: 5’-CCAGGGGTGCCCAGCGAATG-3’
- MYOGENIN REV: 5’-AGCCGTGAGCAGATGATCCCC-3’
- MyHC FW: 5’-GCATCGAGCTCATCGAGAAG-3’
- MyHC REV: 5’-CATACAGCTTGTTCTTGAAGGAGG-3’
- DYS49-50 F: 5’- AGCCAGTGAAGAGGAAGTTAGAAG-3’
- DYS50 R: 5’- AATAGTGGTCAGTCCAGGAGCTA-3’
- h actinin F: 5’- CTACAATGAGCTGCGTGTGG -3’
- h actinin R: 5’- TAACCCTCATGTCAGGCAGA -3’
- NV1 F: 5’- ACAGGTTTCTGCTTCTGGCT -3’
- NV1 R: 5’- CATCAGCTGACTGGTTCACA -3’

Appendix References

Araki K, Okada Y, Araki M, Yamamura K (2010) Comparative analysis of right element mutant lox sites on recombination efficiency in embryonic stem cells. BMC biotechnology 10**:** 29

Hiratsuka M, Ueda K, Uno N, Uno K, Fukuhara S, Kurosaki H, Takehara S, Osaki M, Kazuki Y, Kurosawa Y et al (2015) Retargeting of microcell fusion towards

recipient cell-oriented transfer of human artificial chromosome. BMC biotechnology 15**:** 58

Katoh M, Kazuki Y, Kazuki K, Kajitani N, Takiguchi M, Nakayama Y, Nakamura T, Oshimura M (2010) Exploitation of the interaction of measles virus fusogenic envelope proteins with the surface receptor CD46 on human cells for microcell-mediated chromosome transfer. BMC biotechnology 10**:** 37

Kazuki Y, Hiratsuka M, Takiguchi M, Osaki M, Kajitani N, Hoshiya H, Hiramatsu K, Yoshino T, Kazuki K, Ishihara C et al (2010) Complete genetic correction of ips cells from Duchenne muscular dystrophy. Molecular therapy : the journal of the American Society of Gene Therapy 18**:** 386-393

Koi M, Shimizu M, Morita H, Yamada H, Oshimura M (1989) Construction of mouse A9 clones containing a single human chromosome tagged with neomycin-resistance gene via microcell fusion. Jpn J Cancer Res 80**:** 413-418
