# Supplementary material for: Reversible immortalisation enables genetic correction of human muscle progenitors and engineering of next‐generation human artificial chromosomes for Duchenne muscular dystrophy
Source: EMBO Mol Med. 2017 Dec 14;10(2):254–75. doi: 10.15252/emmm.201607284 (PMC5801502; doi:10.15252/emmm.201607284)
Supplement: Supplementary file 2 — Expanded View Figures PDF [file EMMM-10-254-s002.pdf]

Expanded View Figures

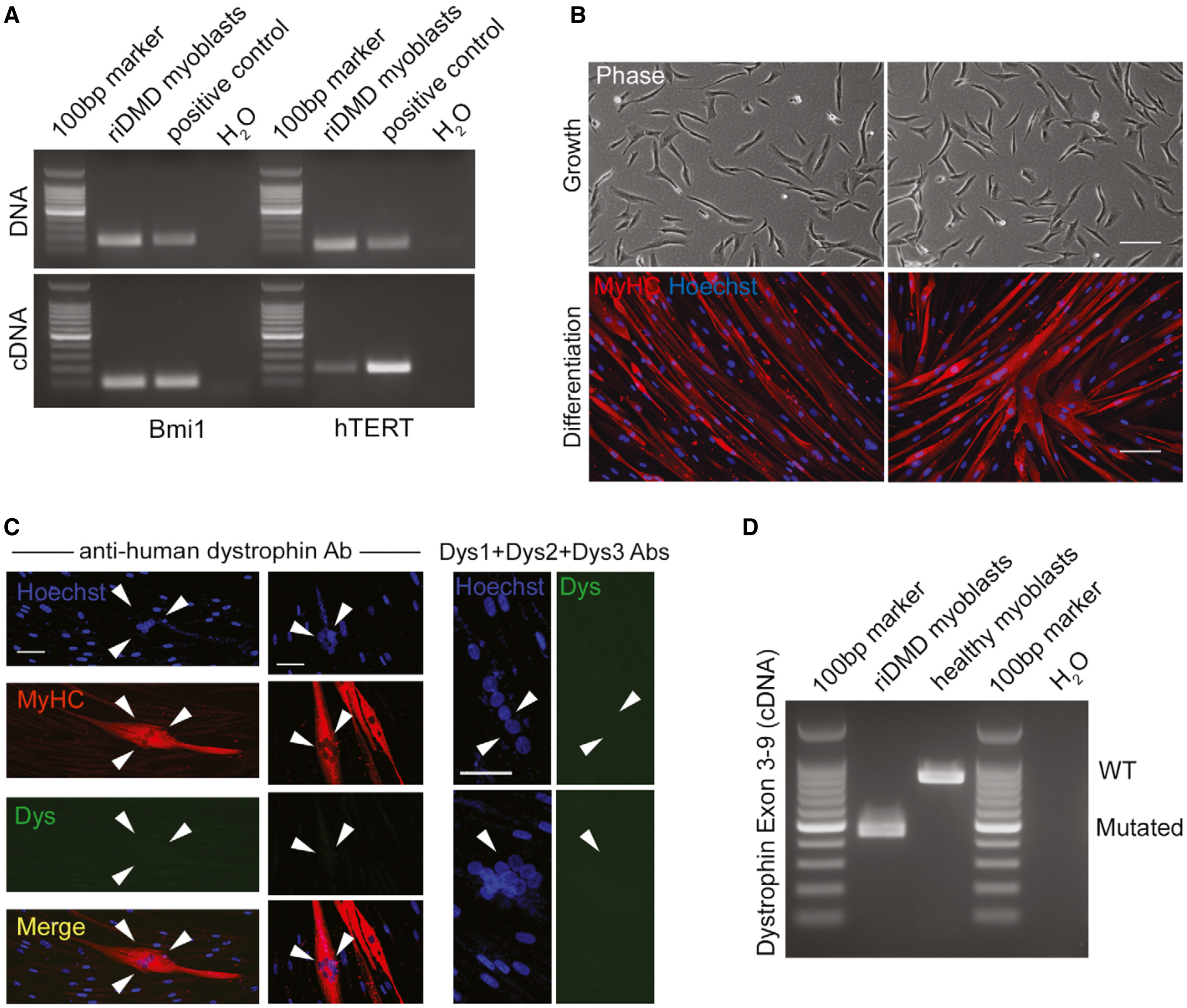

Figure EV1. Characterisation of DMD immortalised (riDMD) myoblasts.

A PCRs for hTERT and Bmi1 on genomic DNA and cDNA of reversibly immortalised myoblasts (riDMD myoblasts). Positive control: immortalised mesoangioblasts.

B riDMD myoblasts in proliferation (phase contrast, upper images) and after myogenic differentiation (lower images). Red: myosin heavy chain (MyHC); blue: Hoechst. Scale bar: 100 μm.

C Dystrophin immunofluorescence in riDMD myoblasts myotubes (white arrowheads). Red: MyHC; green: dystrophin; blue: Hoechst; yellow: merge. Scale bar: 50 μm.

D RT–PCR for dystrophin exon 3–9 transcript in differentiated riDMD myoblasts (deletion exons 5–7) confirming the presence of an out-of-frame DMD mutation and absence of alternative splicing variants (i.e. skipping of exon 8), which could potentially restore the reading frame. Healthy myoblasts: positive control. riDMD myoblast band is approximately 450 bp due to amplification of dystrophin exons 3, 4, 8 and 9, whereas healthy myoblast band is expected to be 833 bp due to amplification of exons 3, 4, 5, 6, 7, 8 and 9.

Source data are available online for this figure.

© 2017 The Authors

Figure EV2. Characterisation of immortalised mesoangioblasts.

A Phase contrast (upper row) and fluorescence (lower row) of GFP H#1 and H#2 polyclonal populations and of GFP #B5 clone (from GFP H#3 polyclonal population). Scale bar: 100 μm.

B Western blot showing Bmi1 expression for hTERT + Bmi1 polyclonal populations (hTERT + Bmi1 H#1 and hTERT + Bmi1 H#2) and untransduced parental population (H#1 and H#2). Gapdh: normaliser.

C Population doubling curves (PD = log*N*/log2; *N* = number of initially plated cells/number of collected cells) of untransduced and hTERT + Bmi1 mesoangioblasts derived from healthy donor #1 (H#1) and #2 (H#1). Data expressed as means ± SEM (n = 2). ***P = 0.0001 (H#1), ***P = 0.0007 (H#2), unpaired two-tailed t-test performed on last time point.

D Bar graph showing proliferation rate of untransduced (on the left of the dashed line) and hTERT + Bmi1 (on the right of the dashed line) mesoangioblasts derived, respectively, from healthy donor #1 (H#1) and healthy donor #2 (H#2). Proliferation rate was assessed as the percentage $BrdU^+$ cells on total number of nuclei. Data are expressed as means ± SEM (n = 2).

E Quantification of TRF assay shown in Fig 3I. Data expressed as means ± SD (n = 3).

F Immunofluorescence analysis of hTERT + Bmi1 clones H#3A, H#3B and H#3C spontaneous (left) and MyoD-ER-mediated (right) skeletal muscle differentiation. Red: myosin heavy chain (MyHC); blue: Hoechst. Scale bar: 50 μm.

Source data are available online for this figure.

© 2017 The Authors

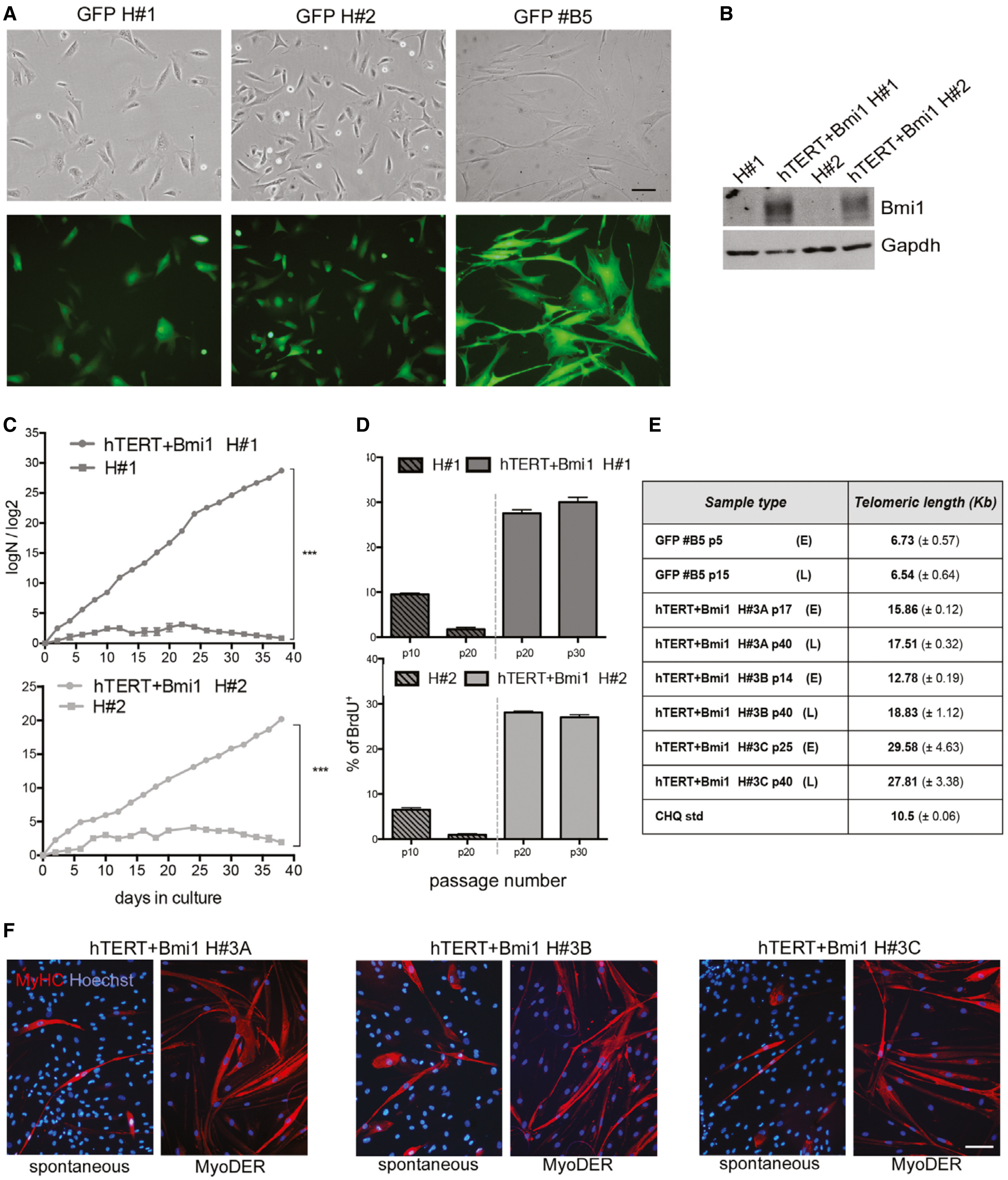

Sample type		*Telomeric length (Kb)*
GFP #B5 p5	(E)	**6.73** (± 0.57)
GFP #B5 p15	(L)	**6.54** (± 0.64)
hTERT+Bmi1 H#3A p17	(E)	**15.86** (± 0.12)
hTERT+Bmi1 H#3A p40	(L)	**17.51** (± 0.32)
hTERT+Bmi1 H#3B p14	(E)	**12.78** (± 0.19)
hTERT+Bmi1 H#3B p40	(L)	**18.83** (± 1.12)
hTERT+Bmi1 H#3C p25	(E)	**29.58** (± 4.63)
hTERT+Bmi1 H#3C p40	(L)	**27.81** (± 3.38)
CHQ std		**10.5** (± 0.06)

Figure EV2.

© 2017 The Authors

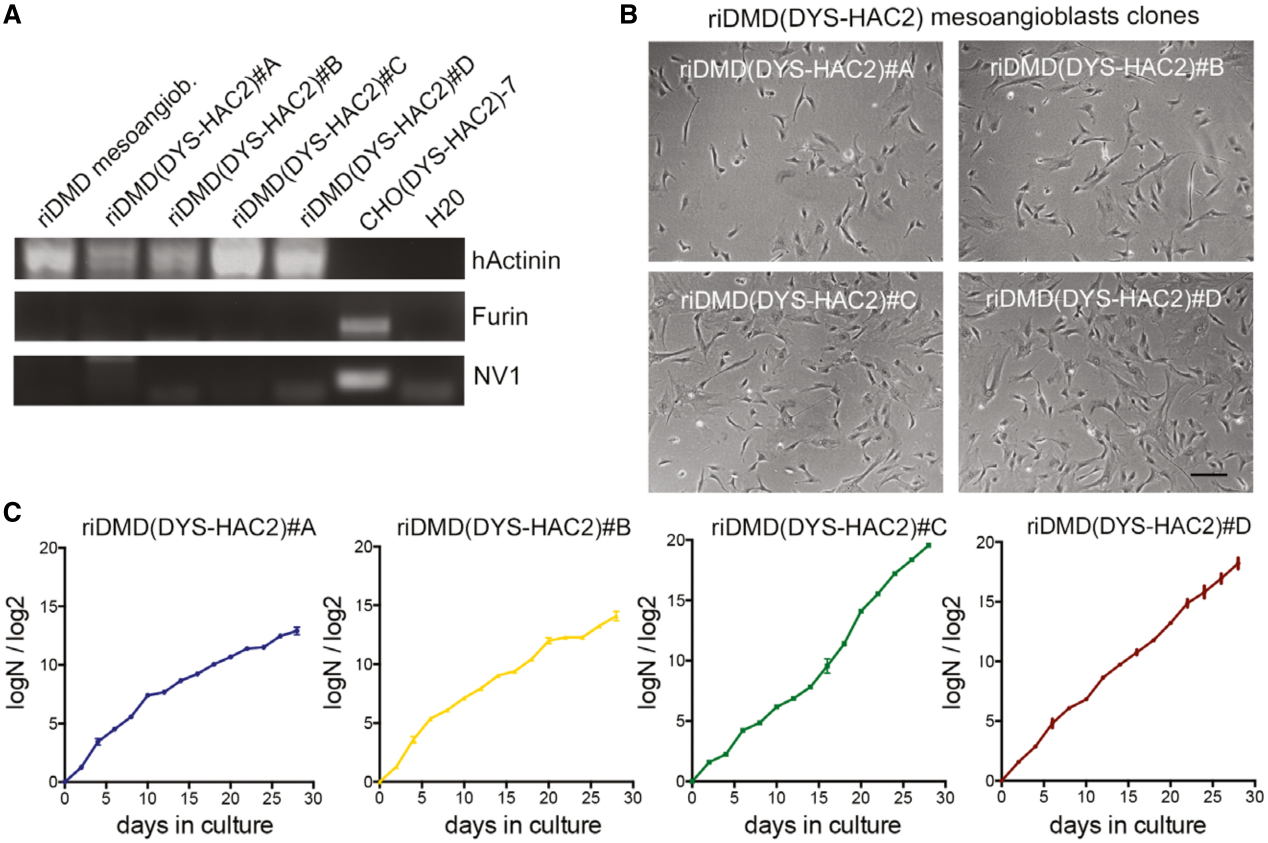

D

DYS-HAC Clone	Initial Number of Cells	Final Number of Cells	Time in Culture
riDMD(DYS-HAC2)#A	3 x 10^4	0,68 x 10^9 (± 0,14 x 10^9)	31 days
riDMD(DYS-HAC2)#B	3 x 10^4	1,66 x 10^9 (± 0.33 x 10^9)	33 days
riDMD(DYS-HAC2)#C	3 x 10^4	23,12 x 10^9 (± 0.64 x 10^9)	31 days
riDMD(DYS-HAC2)#D	3 x 10^4	22,12 x 10^9 (± 0.41 x 10^9)	31 days

Figure EV3. Characterisation of riDMD(DYS-HAC2) mesoangioblast clones.

A PCRs for human actinin, hamster furin and hamster NC1 genes on riDMD(DYS-HAC2) mesoangioblasts clones.

B Phase contrast morphology of riDMD(DYS-HAC2) mesoangioblasts clones riDMD(DYS-HAC2)#A, riDMD(DYS-HAC2)#B, riDMD(DYS-HAC2)#C and riDMD(DYS-HAC2)#D. Scale bar: 100 μm.

C Proliferation curves of riDMD(DYS-HAC) mesoangioblast clones (PD = log*N*/log2; *N* = number of initially plated cells/number of collected cells). Data are expressed as means ± SEM (n = 2).

D Numbers of cells obtained in 31 days of culture for riDMD(DYS-HAC2) mesoangioblast clones starting from 3 × 10^4 plated cells. Data are expressed as means ± SEM (n = 2).

Source data are available online for this figure.

 © 2017 The Authors
