# Supplementary material for: Reversible immortalisation enables genetic correction of human muscle progenitors and engineering of next‐generation human artificial chromosomes for Duchenne muscular dystrophy
Source: EMBO Mol Med. 2017 Dec 14;10(2):254–75. doi: 10.15252/emmm.201607284 (PMC5801502; doi:10.15252/emmm.201607284)
Supplement: Supplementary file 3 — Table EV1 [file EMMM-10-254-s003.pdf]

Table EV1. Tumorigenic assays.

	Sample name	*No. of mice injected*	*Tumours*	*Outcome*
Immortalised mesoangioblasts	hTERT+Bmi1 H#1 (polyclonal)	4	None	0 out of 4
	hTERT+Bmi1 H#2 (polyclonal)	4	None	0 out of 4
	hTERT+Bmi1 H#3A (clone)	4	None	0 out of 4
	hTERT+Bmi1 H#3B (clone)	4	None	0 out of 4
	hTERT+Bmi1 H#3C (clone)	4	None	0 out of 4
riDMD mesoangioblasts	riDMD mesoangioblasts (polyclonal)	3	None	0 out of 3
	riDMD(DYS-HAC2)#A (clone)	6	None	0 out of 6
	riDMD(DYS-HAC2)#B (clone)	6	None	0 out of 6
	riDMD(DYS-HAC2)#C (clone)	8	None	0 out of 8
	riDMD(DYS-HAC2)#D (clone)	7	None	0 out of 7
riDMD myoblasts	riDMD myoblasts (polyclonal)	3	None	0 out of 3
	riDMD(DYS-HAC2)#α (clone)	5	None	0 out of 5
	riDMD(DYS-HAC2)#γ (clone)	5	None	0 out of 5
	All immortalised cells	TOT 63	None	0 out of 63
	All DYS-HAC2 cells	TOT 37	None	0 out of 37
	Human tumorigenic cells (HeLa)	3	Yes	3 out of 3

The table summarises tumorigenic assays performed in this study and divides them in sub-categories: immortalised cell- and DYS-HAC2-corrected cell-injected mice. 2 x 10^6 cells were injected subcutaneously into immunodeficient *scid/beige* mice. Mice were followed up for tumours formation from a minimum of 4 months up to a maximum of 12 months. As a positive control, mice received HeLa cells. In total, 63 mice have been injected with hTERT and Bmi1 immortalised cells and 37 with DYS-HAC2-genetically corrected cells. None of them developed tumours.
