# Supplementary figures and images for: Reversible immortalisation enables genetic correction of human muscle progenitors and engineering of next‐generation human artificial chromosomes for Duchenne muscular dystrophy
Source: EMBO Mol Med. 2017 Dec 14;10(2):254–75. doi: 10.15252/emmm.201607284 (PMC5801502; doi:10.15252/emmm.201607284)

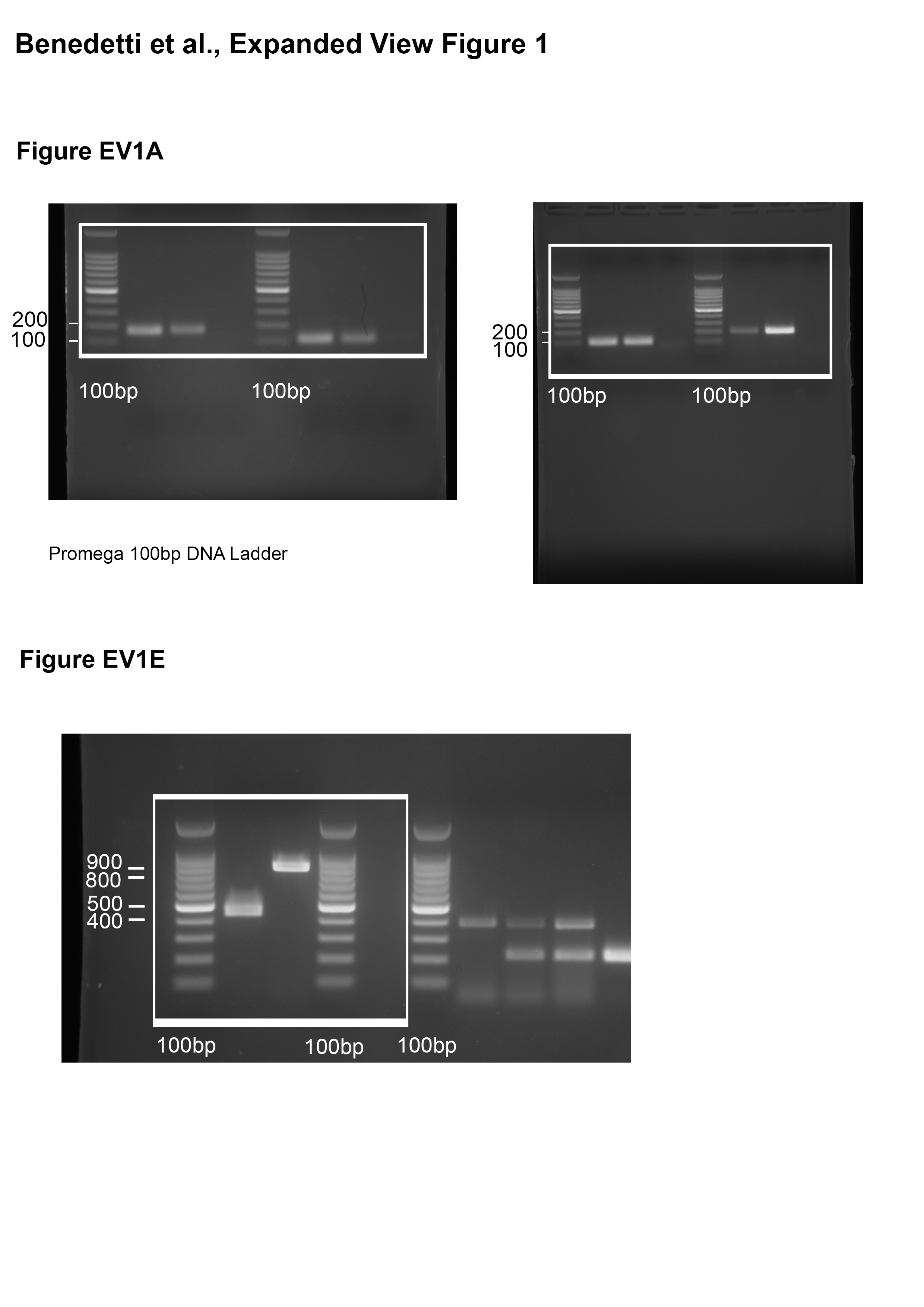

Supplement: Supplementary file 4 — Source Data for Expanded View and Appendix [file EMMM-10-254-s011.zip › Source_Data_EV1.jpg]

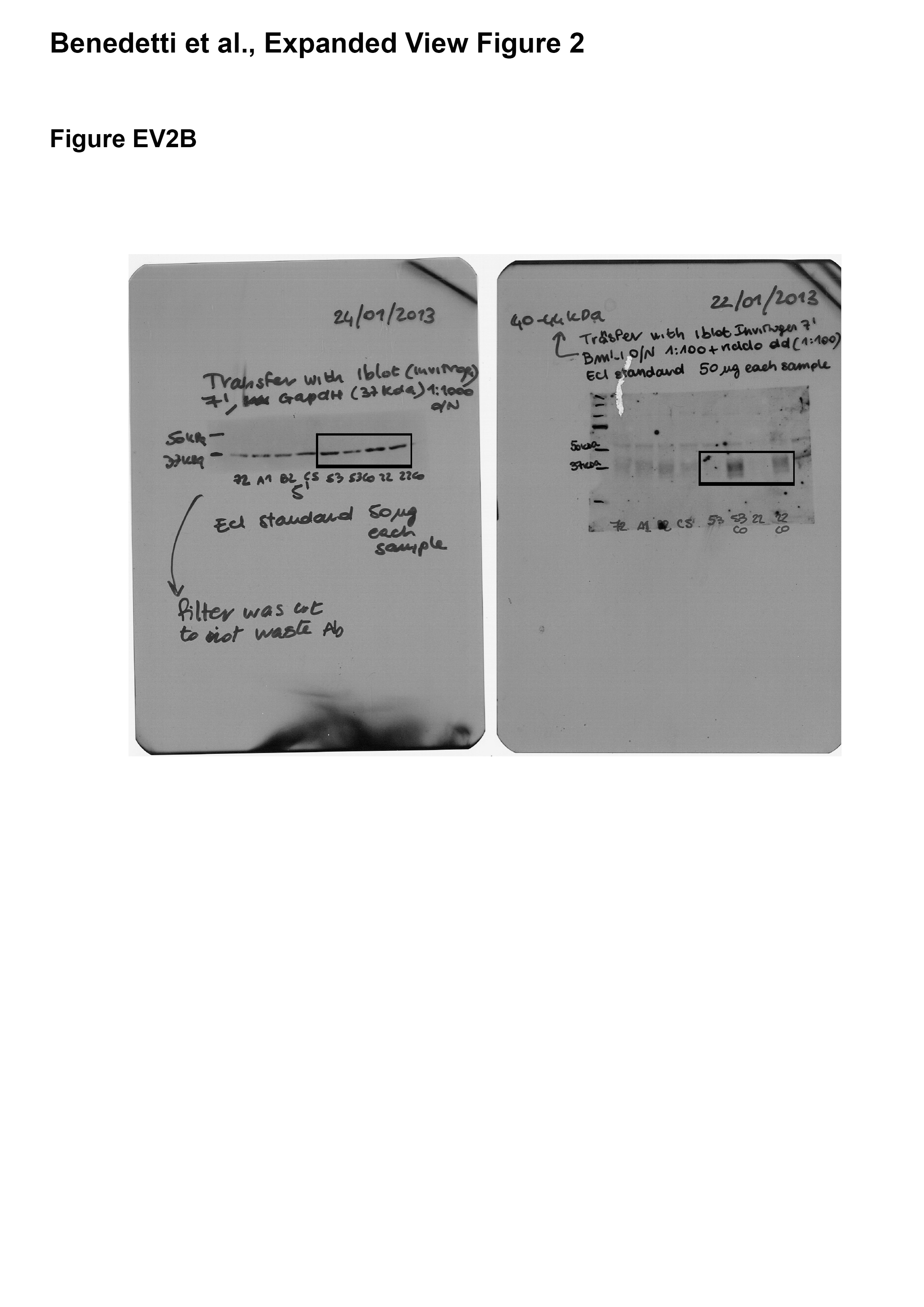

Supplement: Supplementary file 4 — Source Data for Expanded View and Appendix [file EMMM-10-254-s011.zip › Source_Data_EV2.jpg]

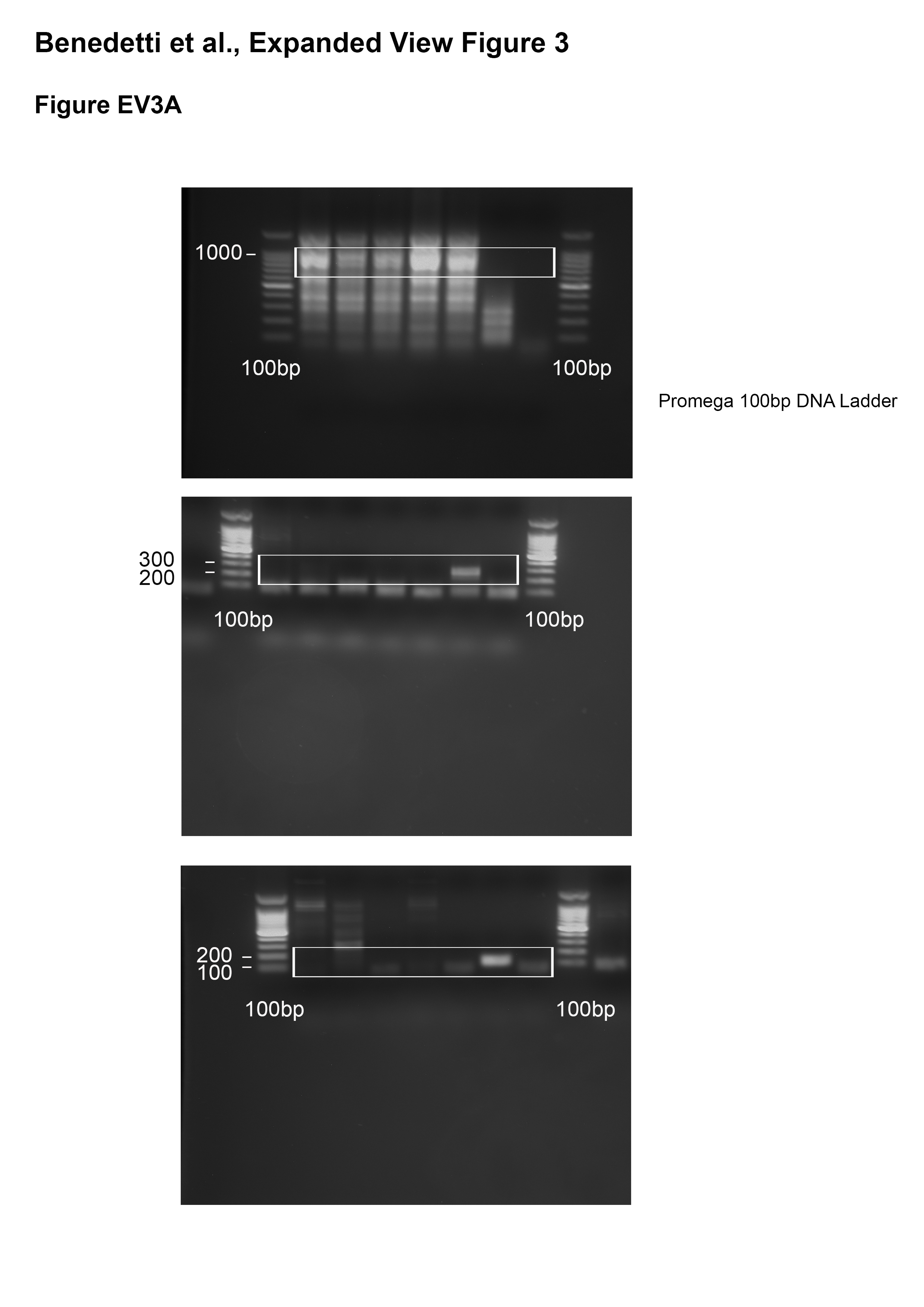

Supplement: Supplementary file 4 — Source Data for Expanded View and Appendix [file EMMM-10-254-s011.zip › Source_Data_EV3.jpg]

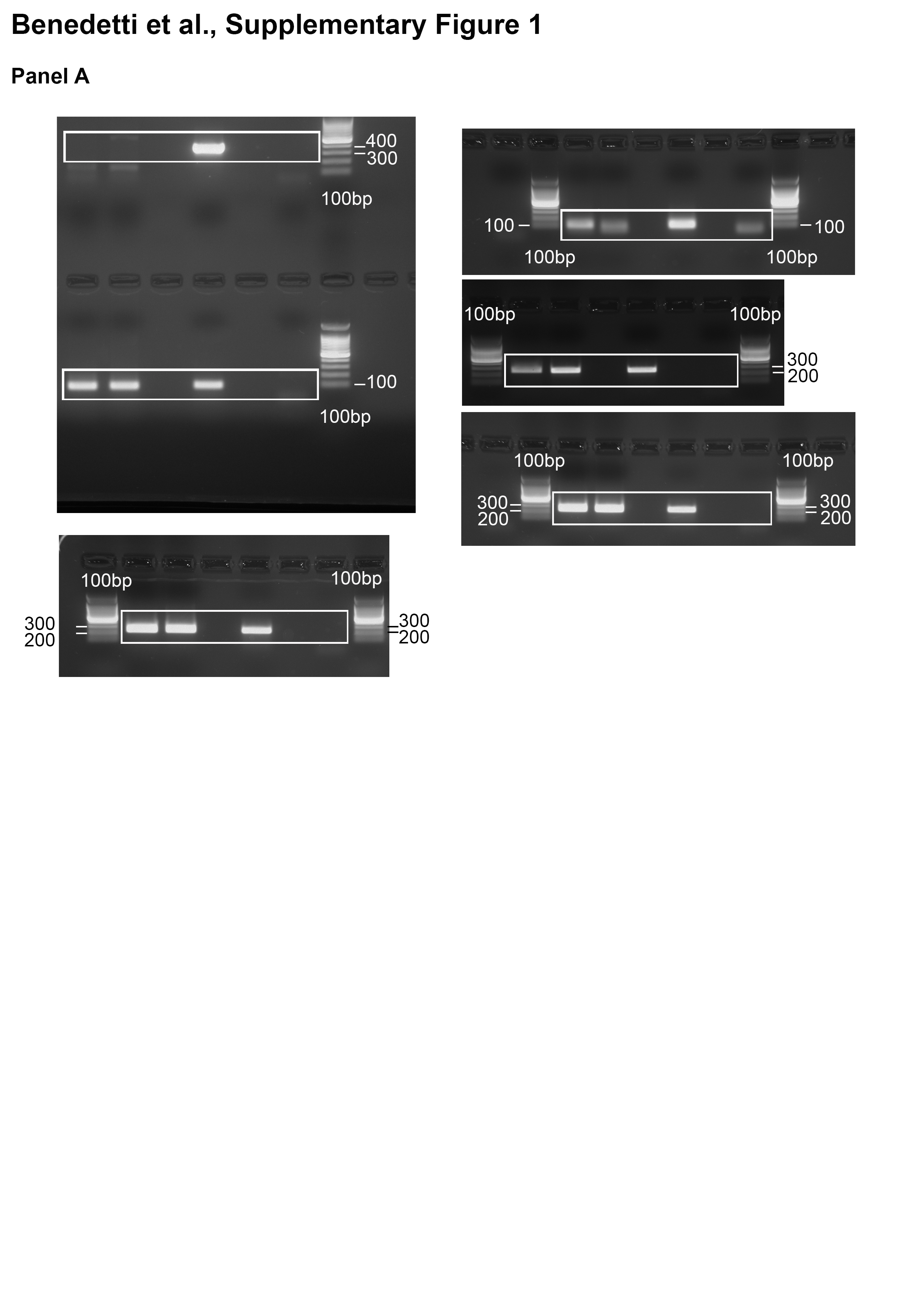

Supplement: Supplementary file 4 — Source Data for Expanded View and Appendix [file EMMM-10-254-s011.zip › Source_Data_S1.jpg]

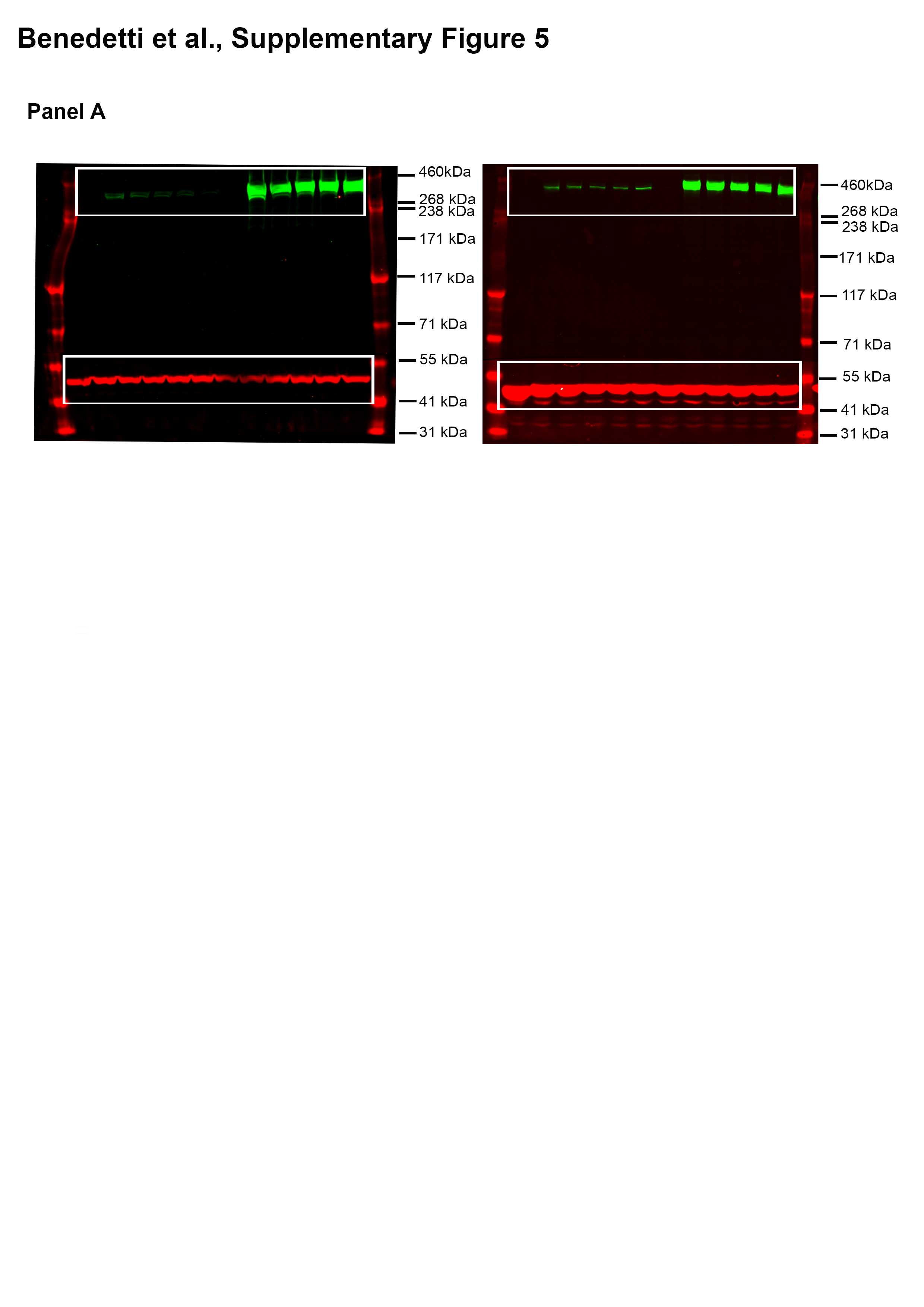

Supplement: Supplementary file 4 — Source Data for Expanded View and Appendix [file EMMM-10-254-s011.zip › Source_Data_S5.jpg]

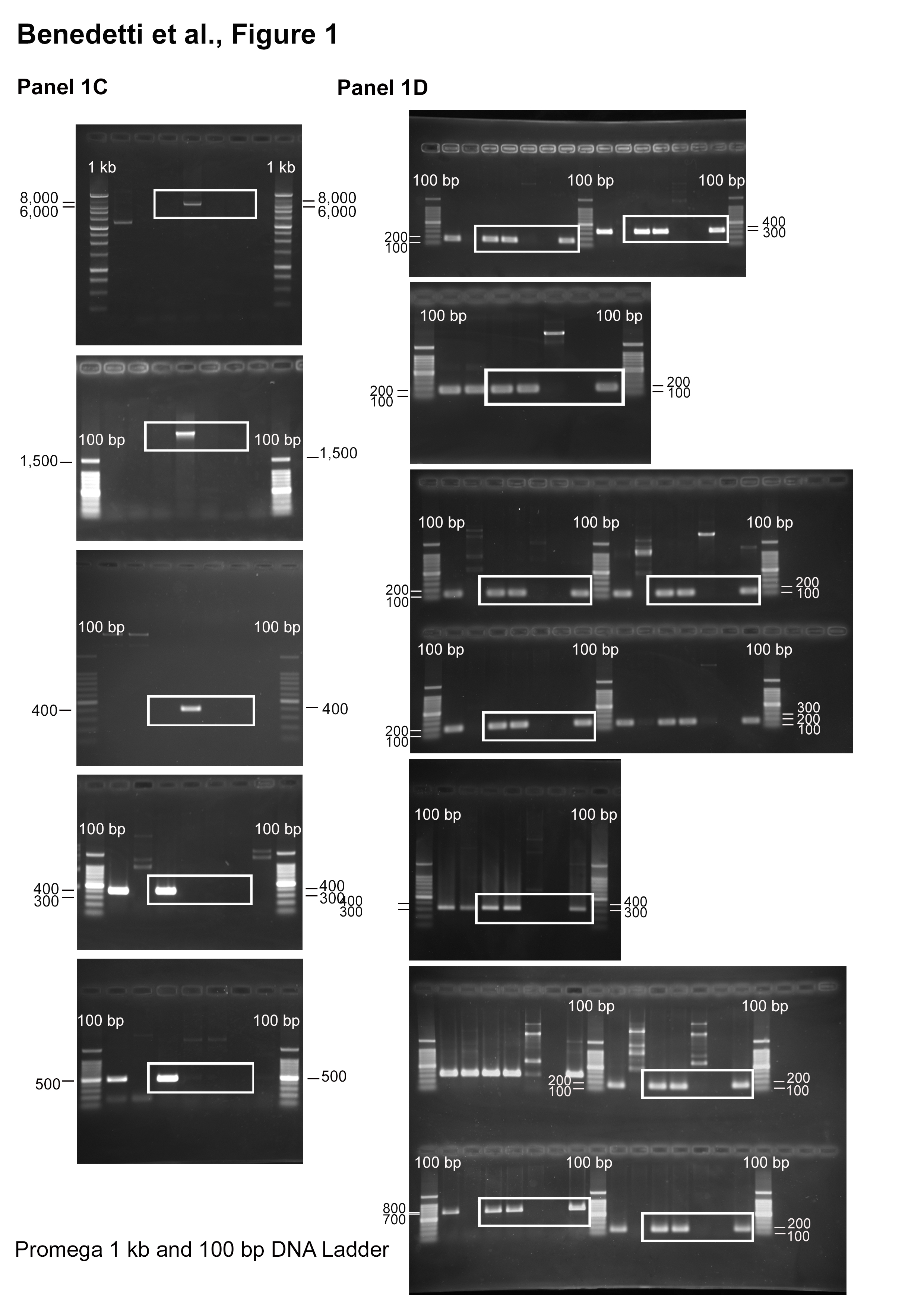

Supplement: Supplementary file 6 — Source Data for Figure 1 [file EMMM-10-254-s004.jpg]

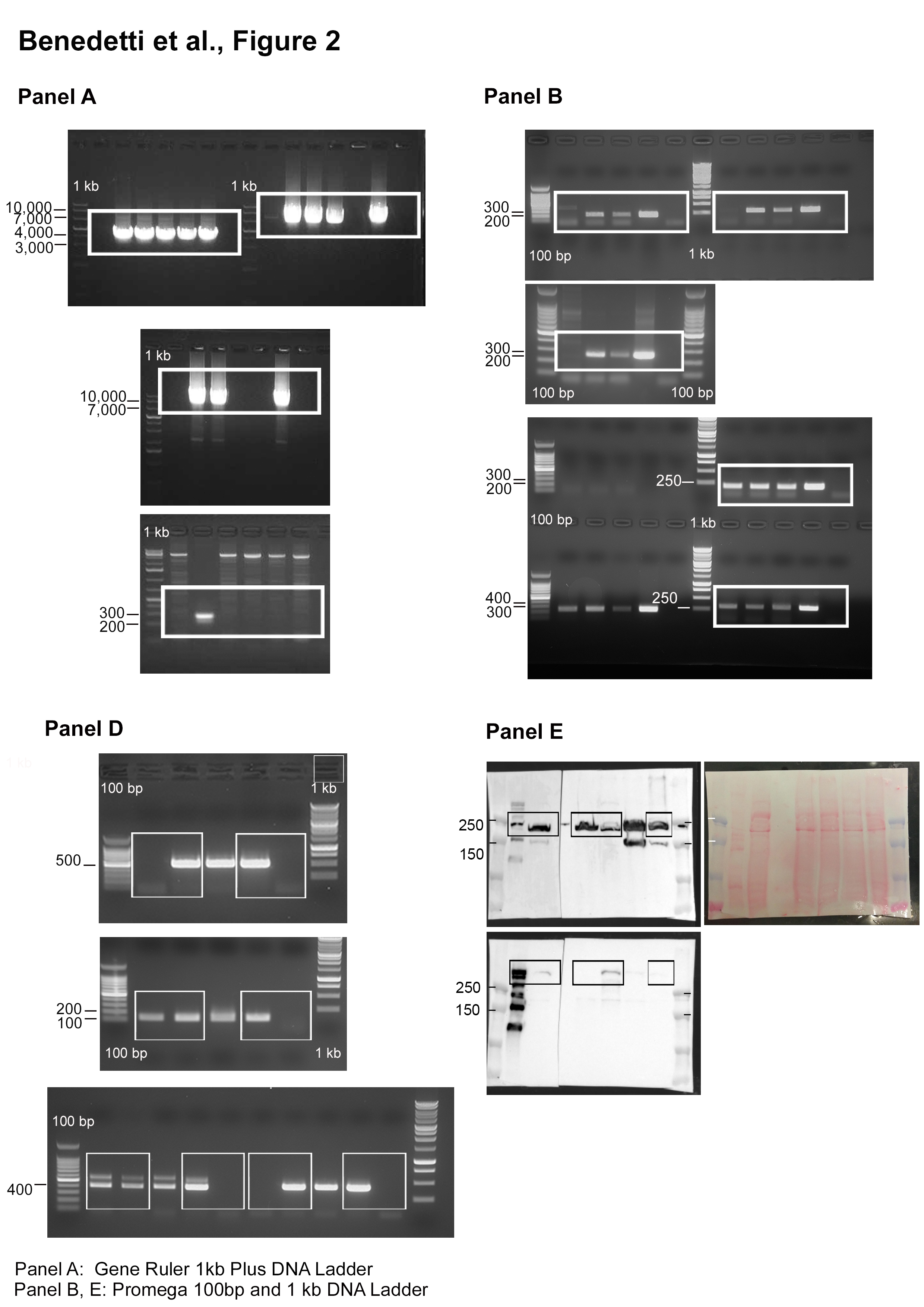

Supplement: Supplementary file 7 — Source Data for Figure 2 [file EMMM-10-254-s005.jpg]

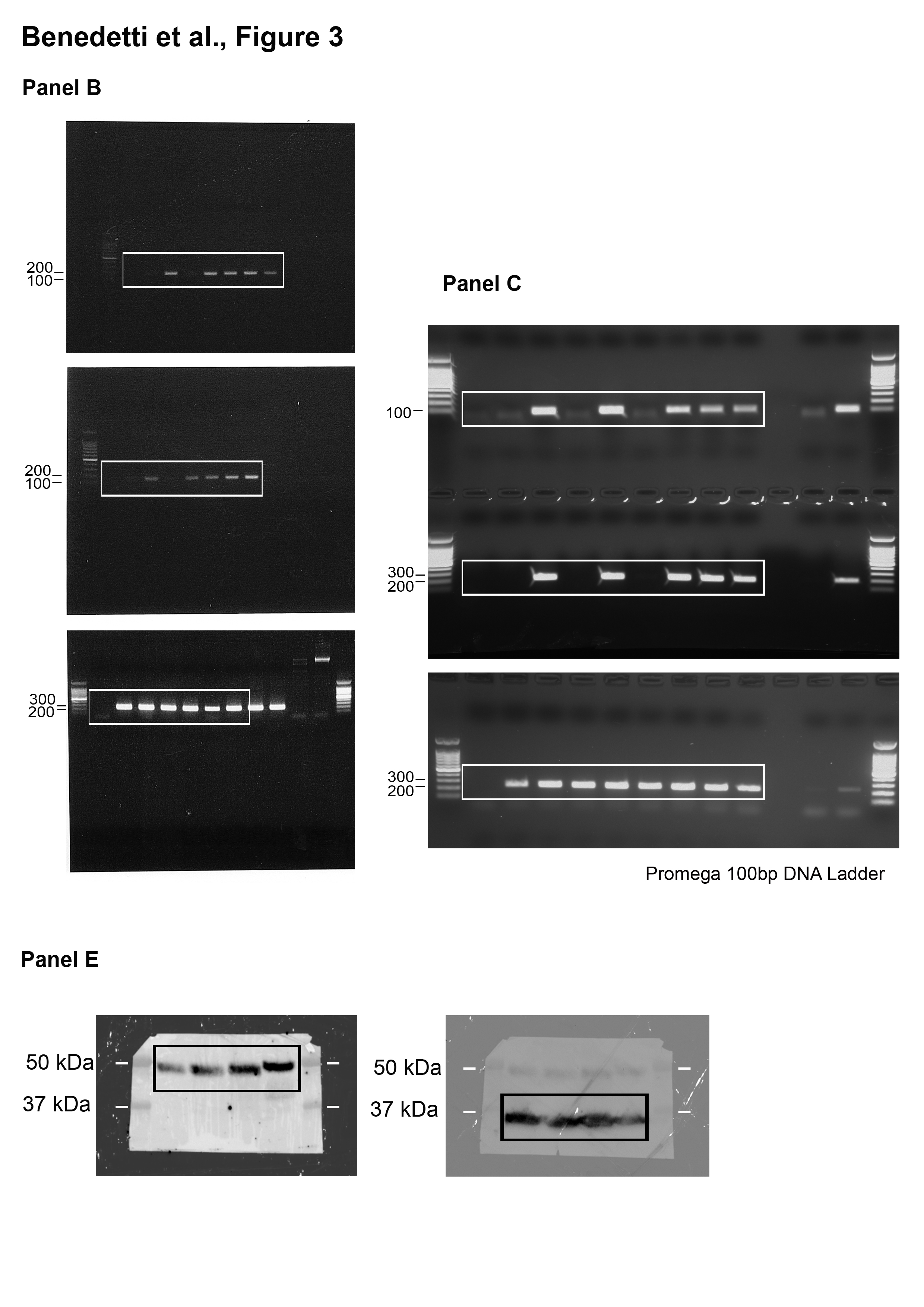

Supplement: Supplementary file 8 — Source Data for Figure 3 [file EMMM-10-254-s006.jpg]

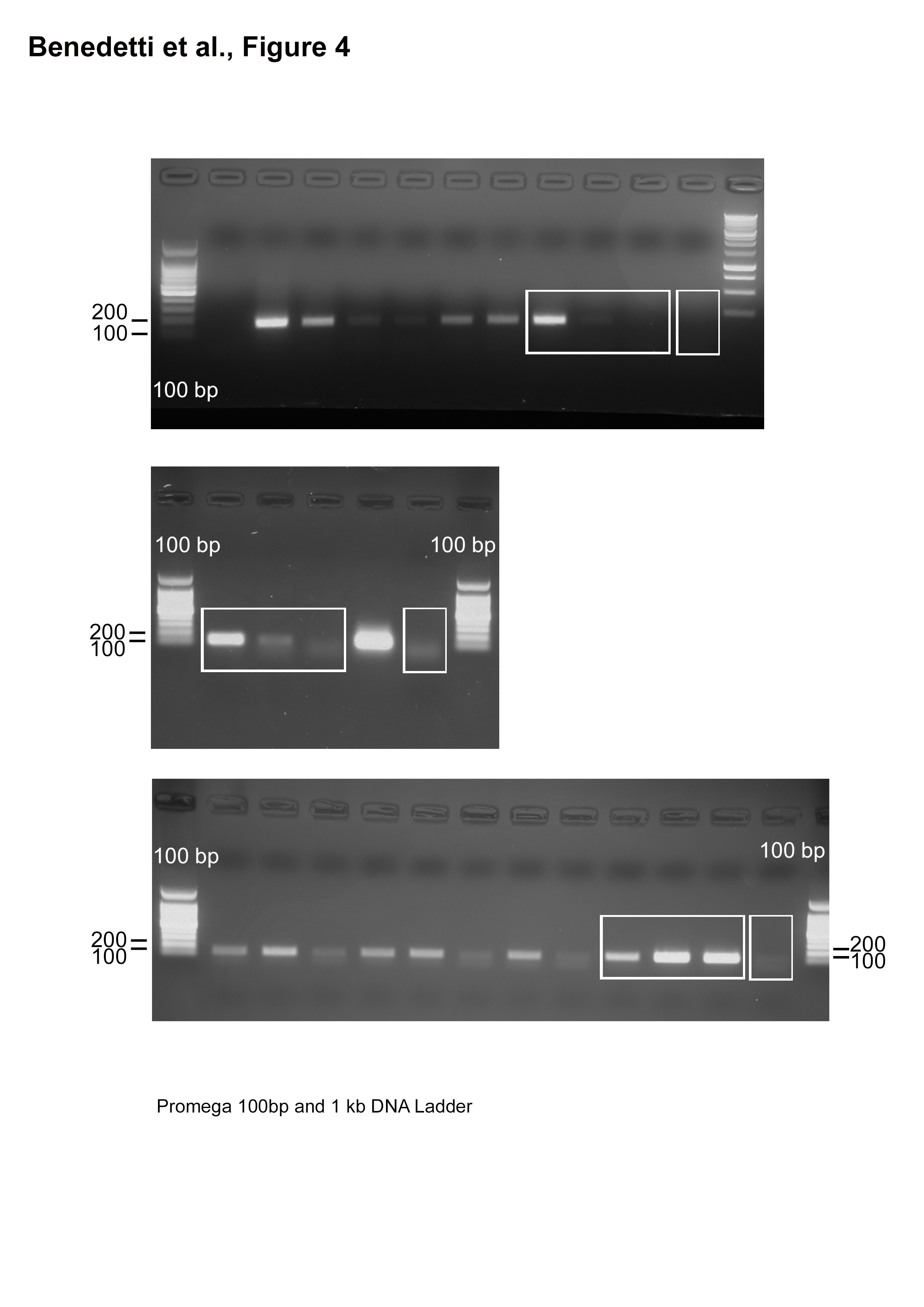

Supplement: Supplementary file 9 — Source Data for Figure 4 [file EMMM-10-254-s007.jpg]

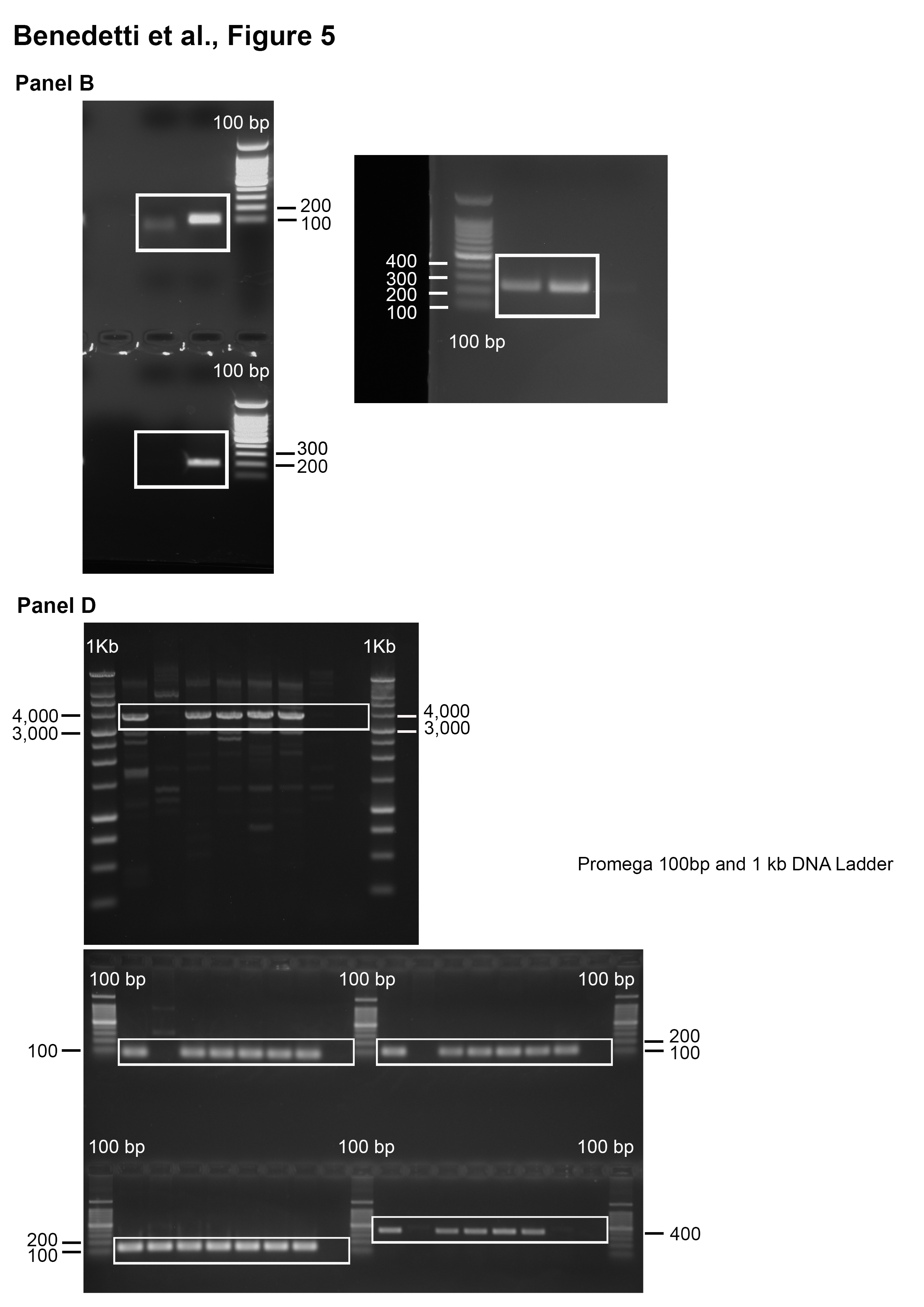

Supplement: Supplementary file 10 — Source Data for Figure 5 [file EMMM-10-254-s008.jpg]

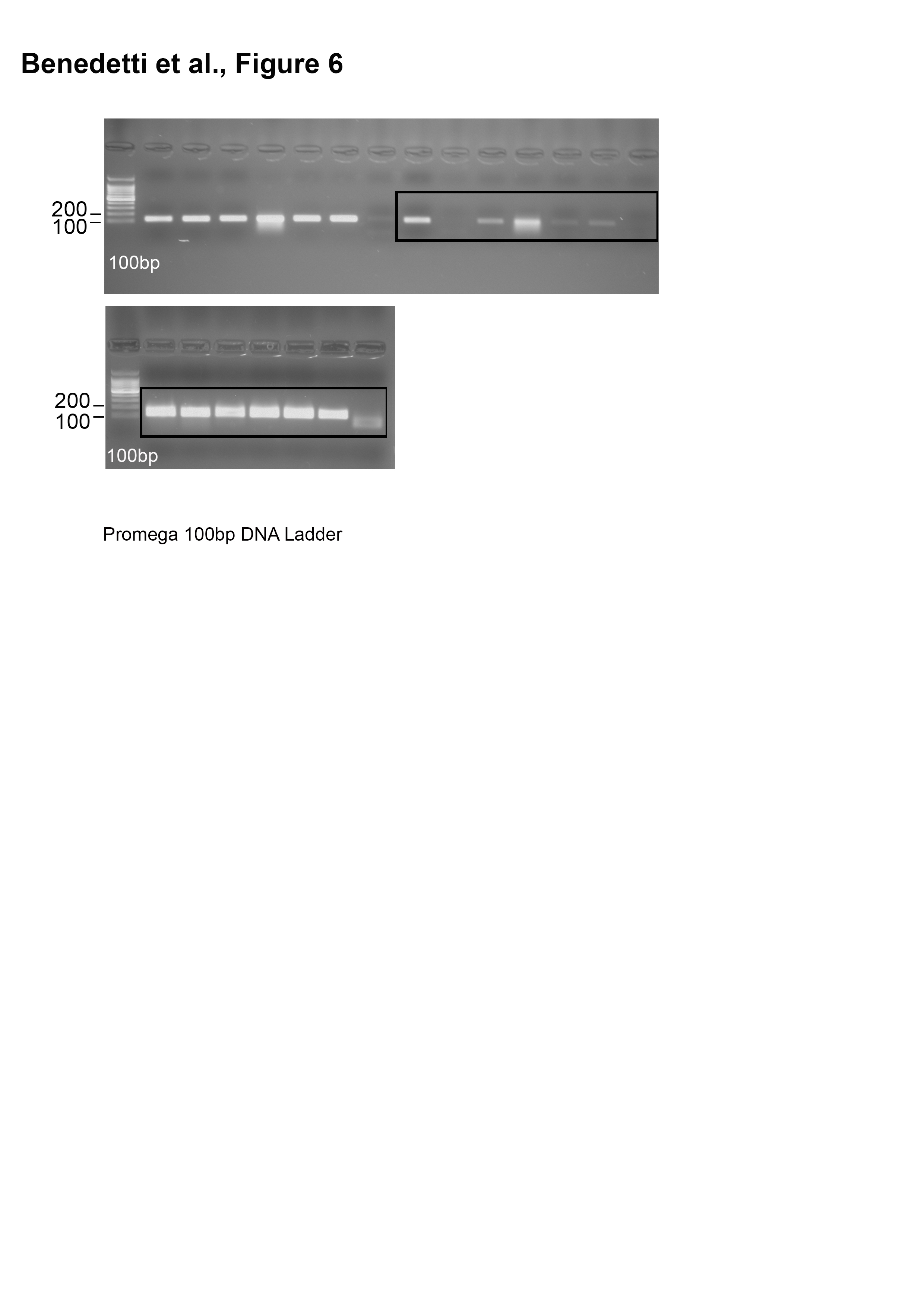

Supplement: Supplementary file 11 — Source Data for Figure 6 [file EMMM-10-254-s009.jpg]
