# Supplementary material for: Reversible immortalisation enables genetic correction of human muscle progenitors and engineering of next‐generation human artificial chromosomes for Duchenne muscular dystrophy
Source: EMBO Mol Med. 2017 Dec 14;10(2):254–75. doi: 10.15252/emmm.201607284 (PMC5801502; doi:10.15252/emmm.201607284)
Supplement: Supplementary file 12 — Source Data for Figure 7 [file EMMM-10-254-s010.pdf]

Benedetti et al., Figure 7, Panel B

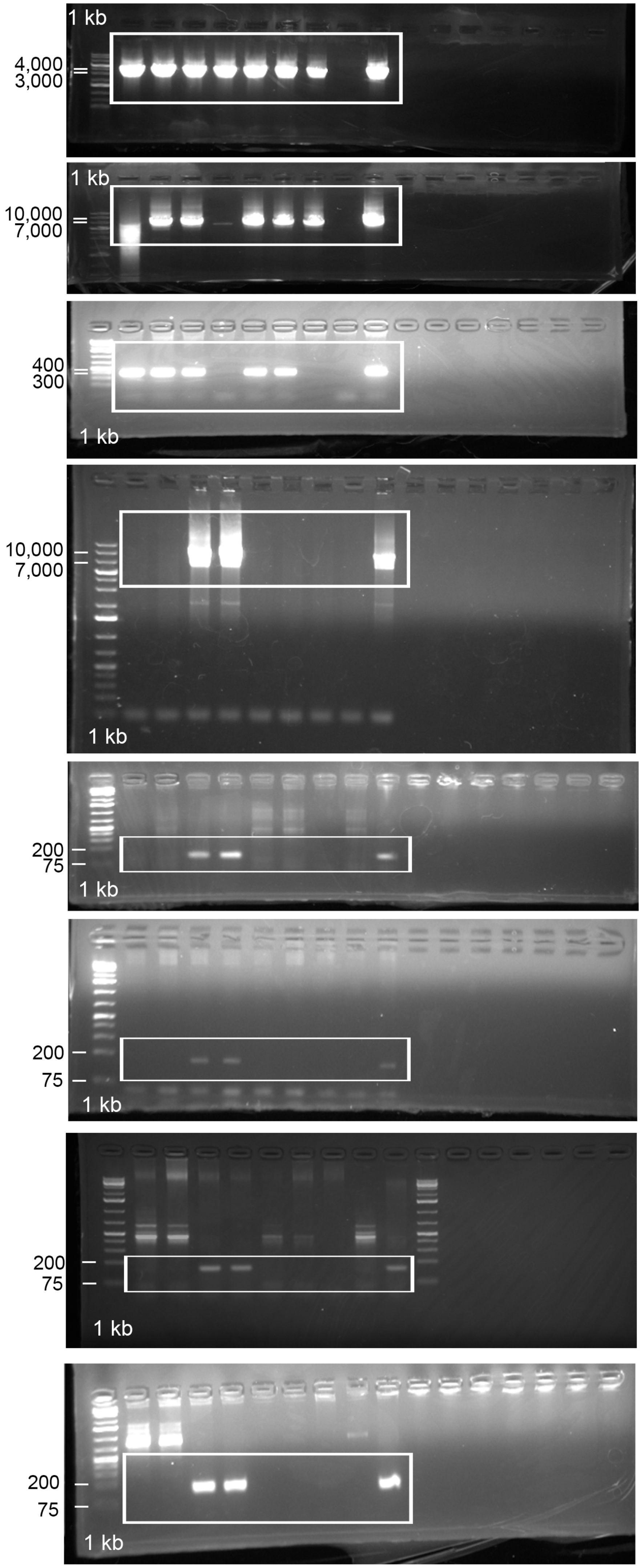

Gene Ruler 1kb Plus DNA Ladder

Benedetti et al., Figure 7, Panel C

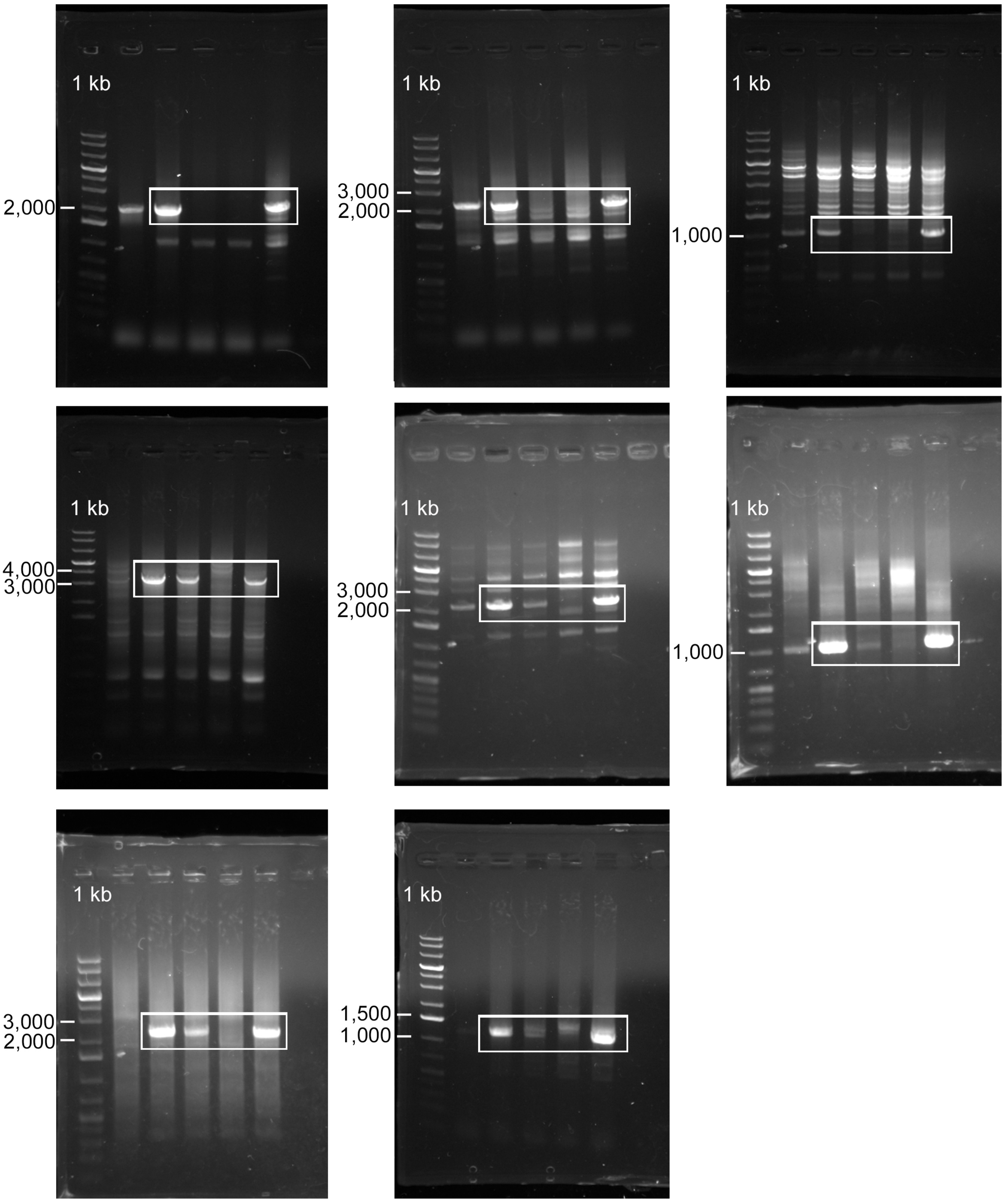

Gene Ruler 1kb Plus DNA Ladder

Benedetti et al., Figure 7, Panel D

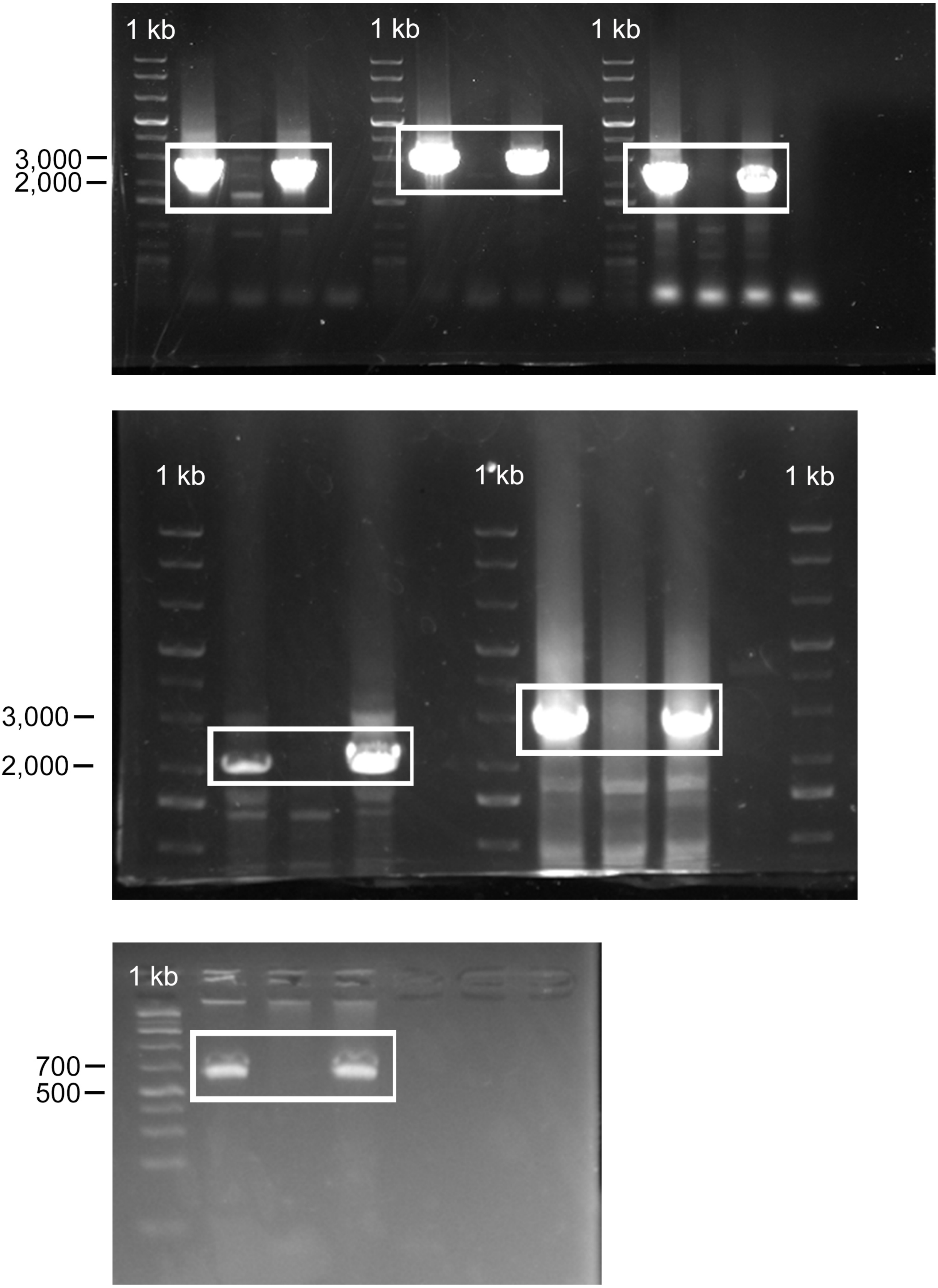

Gene Ruler 1kb Plus DNA Ladder

Benedetti et al., Figure 7, Panel F

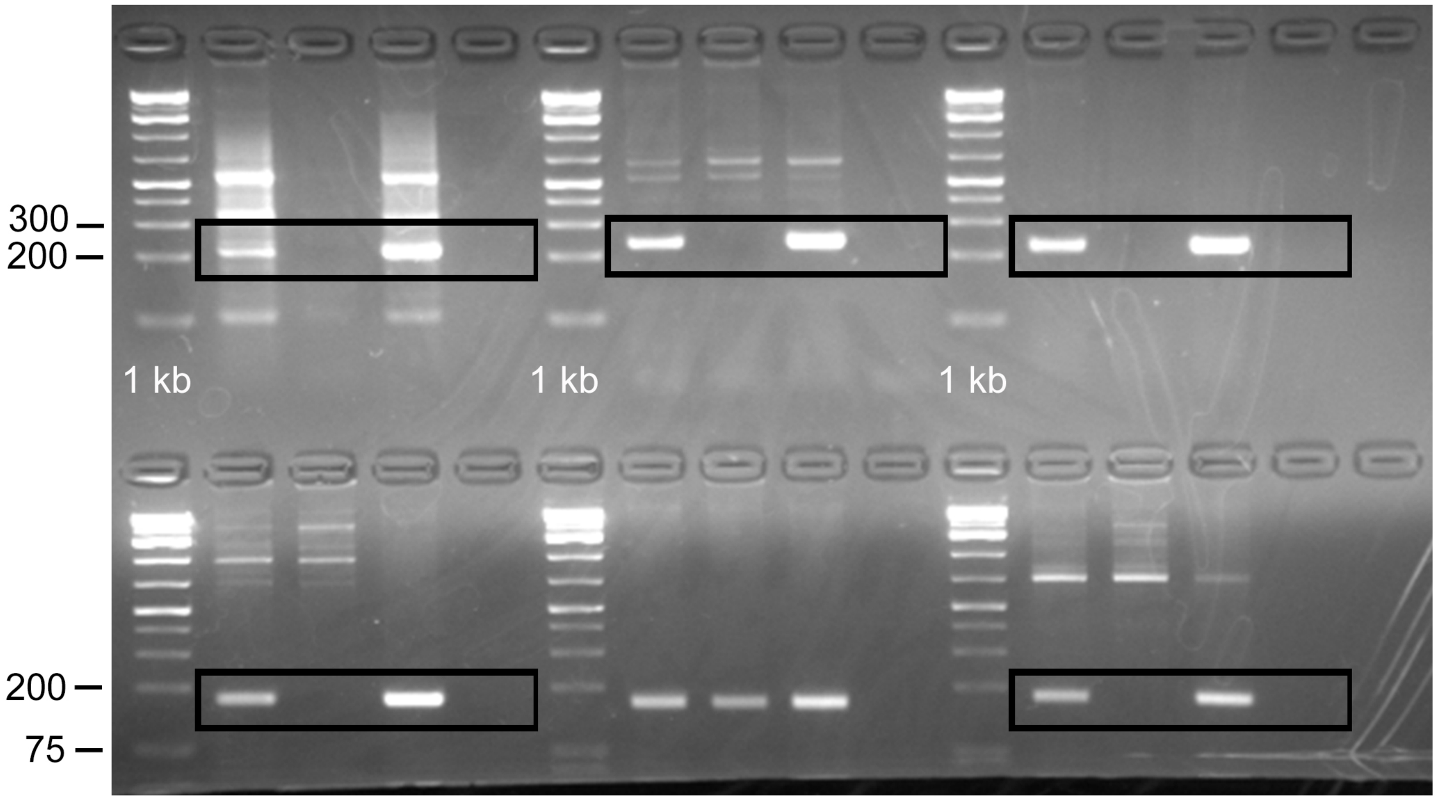

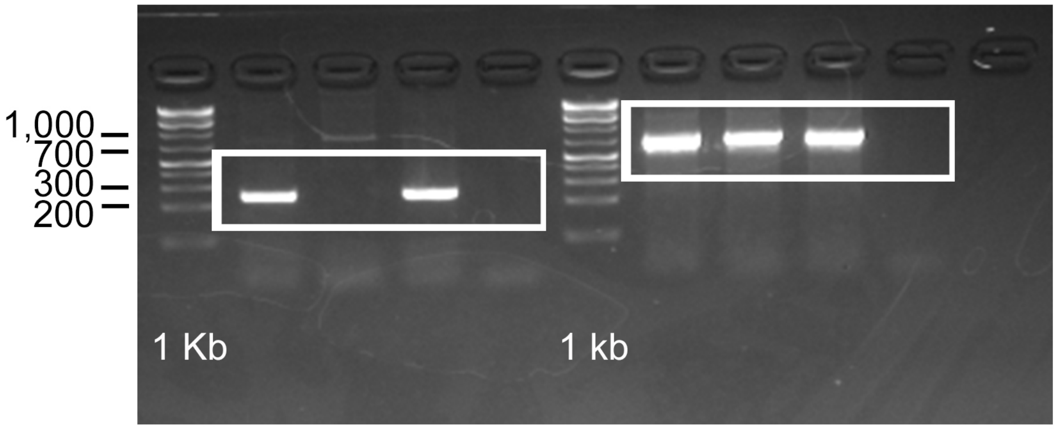

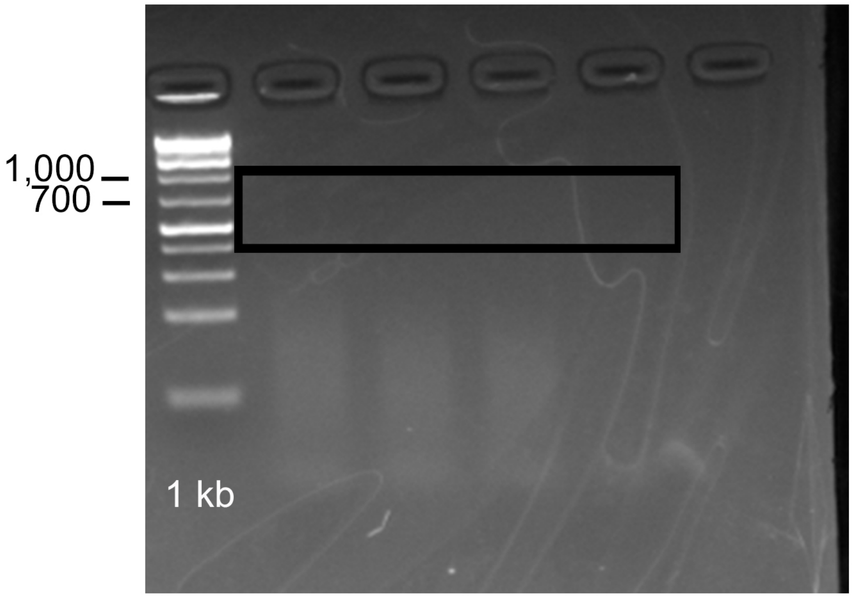

Gene Ruler 1kb Plus DNA Ladder
